# Conformational rearrangements in the sensory RcsF/OMP complex mediate signal transduction across the bacterial cell envelope

**DOI:** 10.1371/journal.pgen.1010601

**Published:** 2023-01-27

**Authors:** Sarah R. Lach, Santosh Kumar, Seonghoon Kim, Wonpil Im, Anna Konovalova

**Affiliations:** 1 Department of Microbiology and Molecular Genetics, McGovern Medical School, The University of Texas Health Science Center at Houston (UTHealth), Houston, Texas, United States of America; 2 The University of Texas MD Anderson UTHealth Graduate School of Biomedical Sciences, Houston, Texas, United States of America; 3 Departments of Biological Sciences, Chemistry, Bioengineering, and Computer Science and Engineering, Lehigh University, Bethlehem, Pennsylvania, United States of America; 4 School of Computational Sciences, Korea Institute for Advanced Study, Seoul, Republic of Korea; Michigan State University, UNITED STATES

## Abstract

Timely detection and repair of envelope damage are paramount for bacterial survival. The Regulator of Capsule Synthesis (Rcs) stress response can transduce the stress signals across the multilayered gram-negative cell envelope to regulate gene expression in the cytoplasm. Previous studies defined the overall pathway, which begins with the sensory lipoprotein RcsF interacting with several outer membrane proteins (OMPs). RcsF can also interact with the periplasmic domain of the negative regulator IgaA, derepressing the downstream RcsCDB phosphorelay. However, how the RcsF/IgaA interaction is regulated at the molecular level to activate the signaling in response to stress remains poorly understood. In this study, we used a site-saturated mutant library of *rcsF* to carry out several independent genetic screens to interrogate the mechanism of signal transduction from RcsF to IgaA. We analyzed several distinct classes of *rcsF* signaling mutants, and determined the region of RcsF that is critically important for signal transduction. This region is bifunctional as it is important for RcsF interaction with both IgaA and OMPs. The mutant analysis provides strong evidence for conformational changes in the RcsF/OMP complex mediating signal transduction to IgaA, and the first direct evidence that OMPs play an important regulatory role in Rcs signaling.

## Author summary

Regulator of Capsule Synthesis (Rcs) is a signaling pathway that can detect damage in the most outer layers of the bacterial cell, and transmits the information inside the cell to induce a protective response. The Rcs signaling mechanism is poorly understood, especially the early steps of signal transduction that regulate activity of downstream phosphorelay. In this study, we show how the Rcs system relies on changes in how protein components interact, similar to a domino effect. In response to stress, the sensor protein RcsF, partnered with outer membrane proteins, undergoes conformational changes that facilitate its access to the signaling inhibitor IgaA, releasing the brakes and allowing signaling to proceed inside the cell.

## Introduction

The bacterial cell envelope is constantly exposed to varying environmental conditions and is a target for biological assaults, including protein toxins, toxic metabolites, host immune factors, and antibiotics [1]. Bacterial survival under these changing conditions depends on timely detection and repair of envelope damage, and this function is fulfilled by signaling pathways collectively known as envelope stress responses [1]. Envelope stress responses in gram-negative bacteria are very complex because of the multilayered envelope structure harboring the outer membrane (OM) [2]. The OM and the cytoplasmic or inner membrane (IM) enclose a periplasmic compartment containing the peptidoglycan cell wall. Signal transduction across the IM typically relies on Histidine-Aspartate phosphorelays [3]. However, signal transduction across the OM and periplasm presents a fundamental challenge as there is no ATP in the periplasm to allow for protein phosphorylation, and these early steps of signaling remain poorly understood for most envelope stress responses.

The Regulator of Capsule Synthesis (Rcs) is one of the most complex envelope stress responses in bacteria, and is highly conserved in the group of Enterobacteriaceae [4]. Many conditions induce Rcs, including those that damage the peptidoglycan cell wall [5–7] and the OM, specifically its lipopolysaccharide (LPS)-packed outer leaflet [8–11]. Signal transduction across the OM and periplasm relies on poorly understood changes in protein-protein interactions between the OM sensory lipoprotein RcsF and the IM negative regulator IgaA, that controls the activity of the RcsCDB phosphorelay [4].

Mature RcsF consists of several domains [12,13]: a proline-rich N-terminal domain (NTD) (residues 16–48) with a lipidated N-terminal cysteine (residue 16) that is predicted to be disordered, and a C-terminal domain (CTD, residues 49–134), the structure of which has been solved. Within the CTD, there are two distinct subdomains: the highly structured core domain (residues 63–134) stabilized by two non-consecutive disulfide bonds, and the poorly structured region (residues 49–62) that serves as a junction between the NTD and the structured core domain [12,13].

**Fig 1:**
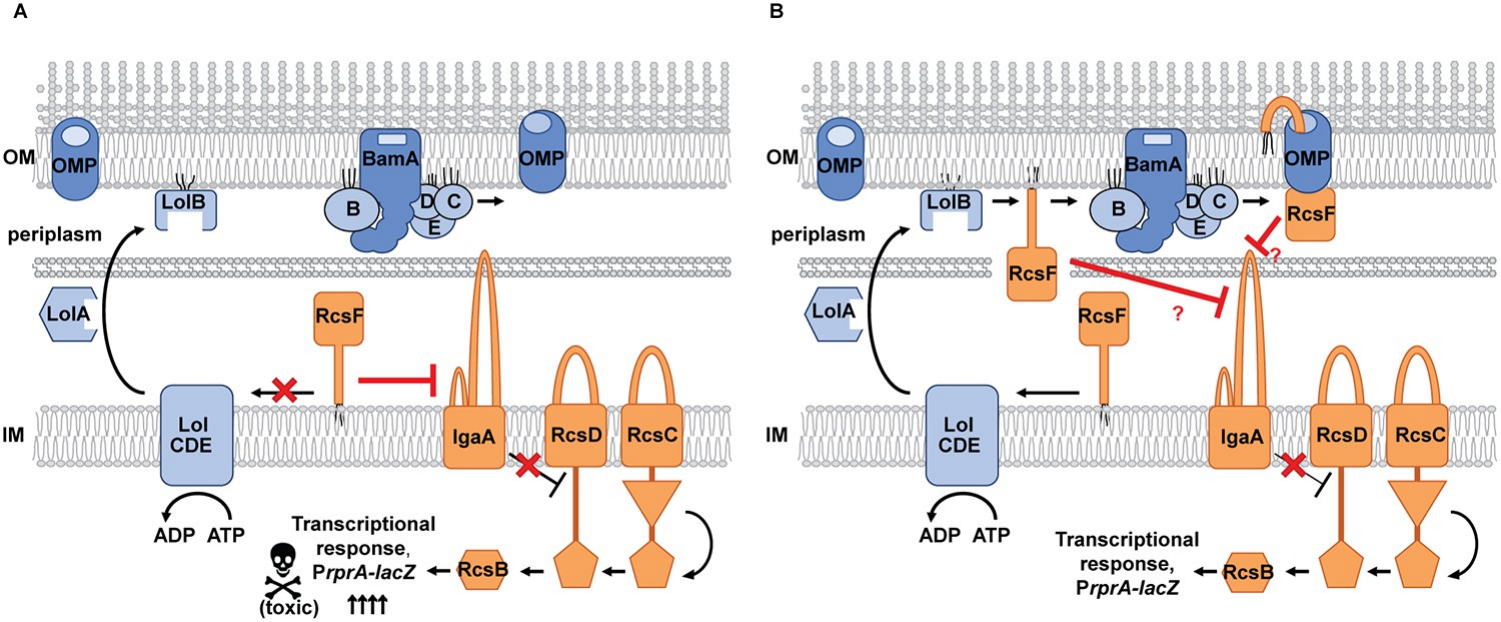
The Rcs stress response pathway. The IM hybrid histidine protein kinase RcsC and the phosphotransferase protein RcsD control the activity of the cytoplasmic DNA-binding response regulator RcsB via phosphorylation. IgaA is an essential negative regulator of the stress response. RcsF is a sensory OM lipoprotein that alleviates IgaA inhibition of the phosphorelay in response to stress. (A) Defects in lipoprotein lipid modification, signal sequence processing or export by the Lol pathway cause RcsF retention at the IM, where it constitutively interacts with IgaA causing toxic overactivation of the system. (B) Under normal conditions, RcsF is exported to the OM and assembled in a complex with OMPs. How the RcsF/OMP complex signals to IgaA and whether RcsF retention in the inner leaflet of the OM can constitutively activate the system was not resolved before the present study.

When RcsF is mislocalized to the IM or expressed as a soluble periplasmic protein, this causes constitutive deregulated activation of Rcs, which is often toxic to the cell (Fig 1A) [14–17]. In the context of mislocalized RcsF, the NTD is dispensable for function, implicating the CTD as a signaling domain sufficient for the release of IgaA-mediated inhibition of the phosphorelay [13,18,19]. Consistent with the genetic evidence, the CTD was shown to interact directly with the periplasmic domain of IgaA *in vitro* [6,20]. These observations led to a widely accepted Rcs model in which stress response activity is regulated at the level of RcsF CTD availability for IgaA interaction. However, how RcsF initiates interaction with IgaA in response to stress remains largely unknown. One of the reasons is a complicated RcsF biogenesis pathway (Fig 1B). RcsF activity is influenced not only by envelope stress but also by the activity of two essential envelope biogenesis pathways. The localization of lipoproteins (Lol) pathway transports RcsF to the OM, and the β-barrel assembly machinery (Bam) assembles RcsF in a complex with either of several other outer membrane proteins (OMPs), including OmpA, OmpC, and OmpF, following which RcsF adopts partially surface exposed topology [6,21]. Assembly of RcsF/OMP complex is required for signaling [8,22], but whether OMP directly regulates RcsF function or simply serves as a passive vehicle for allowing the RcsF final topology is currently unknown.

Several studies utilized site-specific crosslinking to study RcsF interaction with OMPs under uninduced conditions (S1 Fig) [6,21,23]. Most crosslinking sites were identified at the distal end of the NTD or within the CTD. Based on the crosslinking and topology studies [6,21,23], we proposed the simplest model that can explain most of the observed results. In this model, RcsF spans the lumen of the OMP, adopting an Outside-In orientation with a portion of the CTD housed within the lumen of OMPs (Fig 1B and S1 Fig). Indeed, structural modeling revealed that the lumen of OmpC and OmpF could readily accommodate these regions of RcsF (S1 Fig). While this model derived from the site-specific crosslinking studies also supported the notion of CTD occlusion from IgaA under uninduced conditions, further dissection of the regulatory mechanism remained challenging. This is why the specific regions of the RcsF CTD required for signaling or interaction with IgaA interaction remain unknown, and no mutants altering RcsF interaction with partner proteins have been reported.

In the present study, we carried out several independent genetic screens to interrogate the mechanism of signal transduction from RcsF to IgaA. We utilized a site-saturated mutant library of the RcsF CTD in combination with next-generation sequencing, allowing us to identify in an unbiased way several distinct classes of *rcsF* signaling mutants. Analysis of these mutants supports a model in which conformational changes in the RcsF/OMP complex mediate signal transduction to IgaA, and provides the first direct evidence for an active role of OMPs in regulating Rcs signaling.

## Results

### Isolation of *rcsF* mutants that disrupt signaling at the IM

When RcsF is mislocalized to the IM, for example, when the activity of the Lol pathway is inhibited, it results in toxic overactivation of Rcs [14–17]. To isolate *rcsF* mutants specifically defective in signaling to IgaA, we capitalized on this IM toxicity (Fig 1A and S2 Fig). Importantly, this loss-of-function at the IM (LOF[IM]) screen allows isolation of *rcsF* mutants that disrupt interaction with IgaA irrespectively of RcsF ability to interact with Bam and/or partner OMPs.

We introduced the previously described site-saturated mutant library of RcsF CTD (residues 50–134) [22] into the host strain MG2201 (*ΔlolB*, *Δlpp*, *ΔrcsF*, *pBAD18*::*lolB*) [17]. This strain expresses LolB, an essential component of the Lol pathway, from an arabinose-inducible promoter, and it can grow without arabinose only when *rcsF* is deleted or when downstream Rcs signaling is inactivated [17]. Therefore, we reasoned that mutations that disrupt RcsF signaling to IgaA in this background would also restore growth without arabinose.

**Fig 2:**
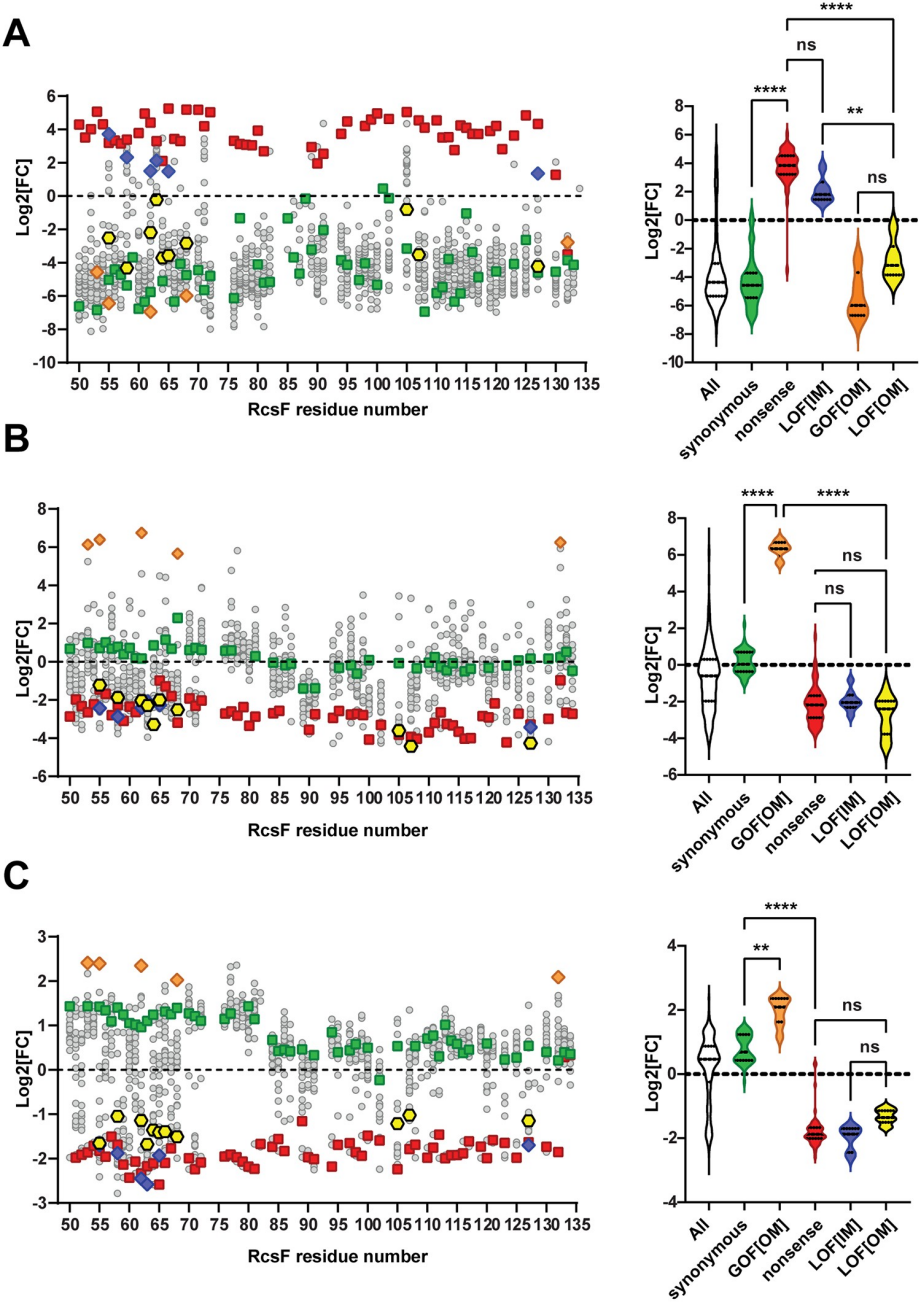
Genetic screens to isolate *rcsF* signaling mutants. (**A**). LOF[IM] screen based on LolB depletion. (**B**) GOF[OM] screen based on the growth on lactose versus glucose (11 generations of outgrowth). (**C**) LOF[OM] screen based on growth on lactose versus glucose (6 generations of outgrowth). Left panels: Log2[FC] of individual RcsF variants plotted against RcsF residue number. Nonsense variants (red), synonymous variants (green), and mutant variants characterized in detail are highlighted as follows: LOF[IM] mutants as blue diamonds, GOF[OM] mutants as orange diamonds, LOF[OM] mutants as yellow hexagons. Right panels: violin plots of log2[FC] distribution within RcsF variant groups. Lines represent the median and the two quartiles. Statistical analysis was performed using one-way ANOVA (multiple comparisons). n.s. = *p* ≥ 0.05, ** *= p* < 0.01, **** *= p* < 0.0001. Complete data for all detected RcsF variants are presented in S1 Dataset.

We subjected mutant pools to growth selection with and without arabinose for approximately 11 generations. After outgrowth, we isolated and sequenced plasmid pools. The Log2 fold change (log2[FC]) in the relative abundance of amino acid (a.a.) variants relative to the “+ arabinose” control was plotted either as individual variants per codon as a scatterplot or groups of variants as a violin plot (Fig 2A). As expected, synonymous mutants encoding wild-type (WT) protein were depleted, while nonsense mutants were enriched across the board except for the two most C-terminal residues. With a log2[FC] cutoff of 1, we identified 58 missense mutants targeting 16 codons (S1 Table). For 7 of these codons, proline was the only a.a. substitution resulting in the phenotype (S1 Table), and we did not pursue these mutants due to the secondary structure-disrupting nature of a proline residue. Remaining mutations were tested for RcsF protein levels, and the phenotypes were reconfirmed in a monoculture (S1 Table). Below, we present phenotypic analysis of representative mutants of six codons. These mutants encoded stable RcsF protein and, most importantly, did not disrupt the interaction of RcsF CTD with BamA in the unrelated screen [21], ruling out the possibility of global disruption of CTD folding as a mechanism of suppression.

### Characterization of *rcsF* mutants that disrupt signaling at the IM

**Fig 3:**
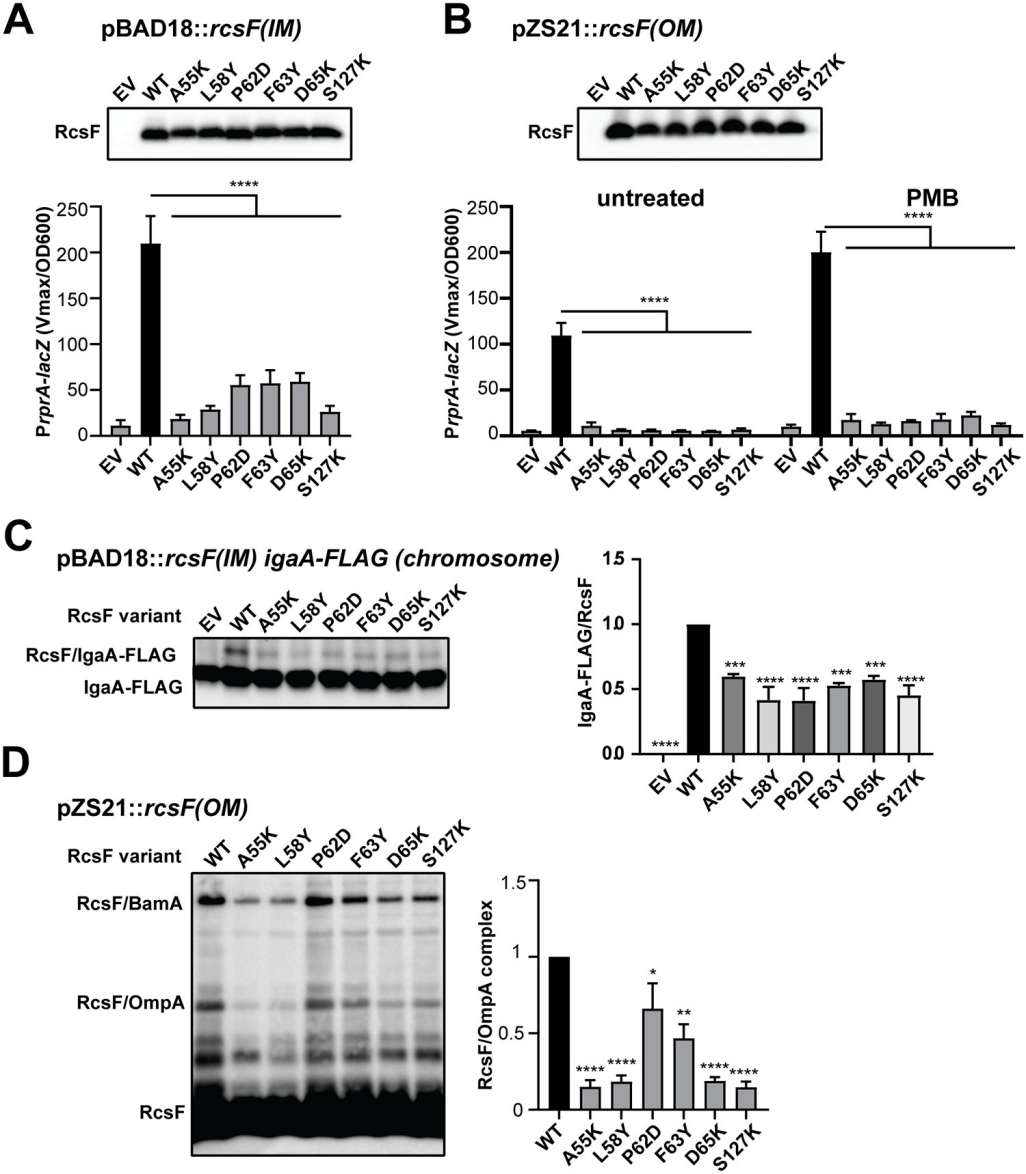
Characterization of RcsF LOF[IM] variants. LOF[IM] mutations were introduced on a plasmid encoding IM **(A)** or OM **(B)** versions of *rcsF* and analyzed by immunoblotting with α-RcsF antibodies and by a β-galactosidase assay using P*rprA-lacZ* transcriptional reporter. Where indicated, strains were treated with 0.75 μg/ml PMB for 40 min. Graphs represent mean β-galactosidase activity normalized to OD600, +/- standard deviation (SD). **(C)** LOF[IM] mutations were introduced into IM localized RcsF and tested for crosslinking to the chromosomally encoded IgaA-FLAG. Immunoblot analysis of *in vivo* formaldehyde crosslinked samples probed with α-FLAG. RcsF/IgaA band was quantified relative to the WT. Graph represents mean of independent experiments +/- SD. **(D)** LOF[IM] mutations affect RcsF/OmpA crosslinking. Immunoblot analysis of *in vivo* formaldehyde crosslinked samples probed with α-RcsF antibodies (left). Immunoblot quantification of RcsF/OmpA relative to the WT (right). Graphs represent mean of three independent experiments +/- SD. Significance throughout this figure is indicated in comparison to the WT as follows: n.s. = *p* ≥ 0.05, * = p < 0.01, ** = p < 0.01, *** = p < 0.001. **** = p < 0.0001. ND = not determined. For additional significance comparison of data (A) and (B) see S2 Table.

To confirm our screen results, we directly tested whether these mutations disrupted signaling at the IM. For this, we introduced an experimentally established Lol-avoidance mutation in the signal sequence (S17D, M18Q [14,21,24]) to retain RcsF at the IM and introduced it into the *ΔrcsF* strain with the *PrprA-lacZYA reporter* fusion [25]. As constitutive RcsF WT expression at the IM is toxic, we employed transient expression from the pBAD18 promoter with a low concentration of arabinose (S3 Fig). Unlike the WT strain, all mutant strains displayed abolished signaling to the level of the empty vector (EV) control (Fig 3A). Next, we tested whether these mutations also inactivated RcsF signaling from the OM. We introduced mutations into *rcsF* encoded on the low copy number vector pZS21. We observed decreased signaling under normal growth conditions and no response to treatment with the LPS-targeting antibiotic polymyxin B (PMB) (Fig 3B and S2 Table).

We next tested whether these mutations disrupted the RcsF/IgaA interaction. For this, we generated a fully functional *igaA-FLAG* fusion at the chromosomal attTn7 site (S4 Fig), introduced pBAD18::*rcsF(IM)* variants, and performed formaldehyde crosslinking. We observed that all RcsF mutant variants showed reduced crosslinking to IgaA (Fig 3C). Crosslinking between OM-tethered RcsF and IgaA is challenging to detect under normal conditions [20]. Therefore, like in previous studies [20], we utilized a plasmid copy of IgaA-FLAG to increase its expression and facilitate detection of the RcsF/IgaA complex. Under these conditions, RcsF mutant variants completely disrupted RcsF/IgaA-FLAG crosslinking (S5 Fig). Remarkably, ColabFold [26] predicted RcsF interaction with the tip of the IgaA periplasmic domain, and this interaction involved the same residues of RcsF as identified using our genetic screen (S6 Fig). The LOF[IM] substitutions are expected to destabilize this RcsF/IgaA interaction based on the *in silico* mutagenesis. Together, our data support the conclusion that these LOF[IM] mutations disrupted signaling by directly disrupting the RcsF/IgaA interaction.

All six residues are located in the same region of RcsF in proximity to previously reported sites of RcsF interaction with OMPs identified by site-specific crosslinking (S7A Fig) [6,21,23], and included one mutation at residue A55 that is important for RcsF/OMP assembly [8]. We, therefore, tested for the ability of these RcsF variants to interact with OMPs at the OM (Fig 3D). Note that we previously showed that the RcsF/BamA complex detected by crosslinking is not an assembly intermediate and not relevant for signaling [22], and therefore we did not focus on it in this study. We used the formation of the RcsF/OmpA complex as a readout because it is readily detectable by the formaldehyde crosslinking in whole cell lysates [8,22,27]. Many of these mutants had significantly (5–10 fold) lower levels of RcsF/OmpA complexes (Fig 3D), demonstrating that this region is functionally important for both IgaA and OmpA interaction, suggesting that the signal may involve a conformational change allowing these regions to switch interacting partners from OMP to IgaA. If this conformational change hypothesis is correct, identifying mutations that favor the RcsF/OMP complex in a constitutively ON or constitutively OFF conformation should be possible.

### Isolation of *rcsF* mutants that alter signaling at the OM

To identify mutations that affect RcsF signaling from the OM (S2 Fig), we introduced the same mutant library into the *ΔrcsF* strain with the *PrprA-lacZYA* reporter (Fig 2B and 2C) and compared growth in M9 minimal media with lactose versus glucose as a sole carbon source. We predicted that gain-of-function at the OM (GOF[OM]) *rcsF* mutants that increase Rcs signaling would increase the expression of the *lacZYA* operon and confer a growth advantage on lactose compared to the WT strain. On the other hand, mutations that decrease RcsF signaling from the OM would prevent growth and cause depletion on lactose, even when compared to the WT variants, as WT strain retains low but detectable *PrprA-lacZYA* expression even under unstressed conditions. We carried out this screen under two different conditions (Fig 2B and 2C). 11 generation outgrowth favors the selection of GOF[OM] mutants, and we identified several mutants that completely outcompeted all other variants (Fig 2B). By contrast, a shorter 6-generation outgrowth allowed reliable distinction between synonymous and nonsense mutants (Fig 2C), aiding in identifying additional signaling defective candidates. As expected, all LOF[IM] variants were also depleted on lactose (Fig 2C), however, we were able to identify mutant candidates that are defective in signaling specifically from the OM (LOF[OM]).

### Characterization of RcsF variants with increased signaling at the OM

**Fig 4:**
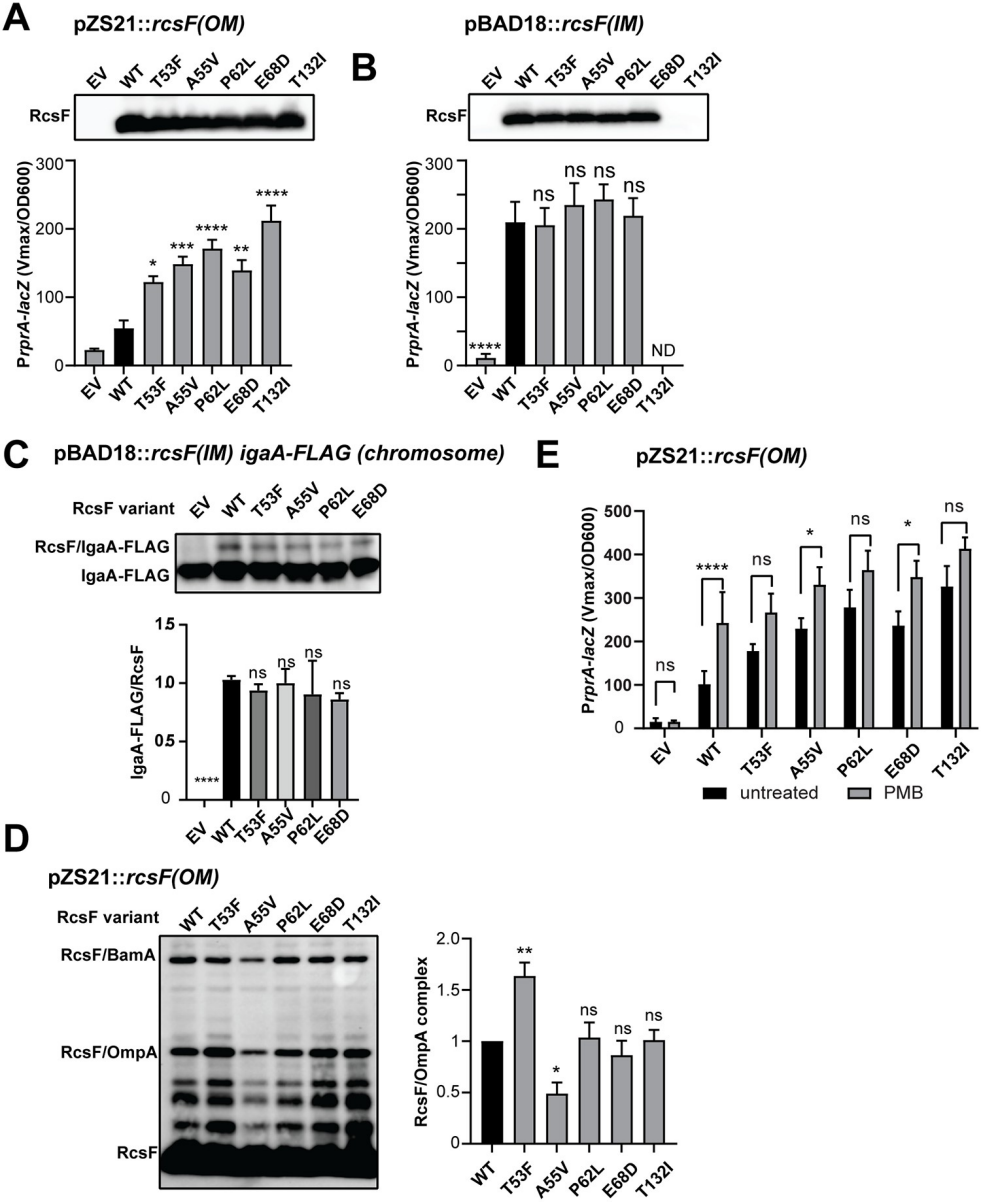
Characterization of RcsF GOF[OM] variants. GOF[OM] mutations were introduced on a plasmid encoding OM **(A)** or IM **(B)** versions of *rcsF* and analyzed by immunoblotting with α-RcsF antibodies and by a β-galactosidase assay using P*rprA-lacZ* transcriptional reporters. Graphs represent mean β-galactosidase activity normalized to OD600 +/- SD. **(C)** GOF[OM] mutations were introduced into the IM localized RcsF and tested for crosslinking to the chromosomally encoded IgaA-FLAG. The quantification of RcsF/IgaA-Flag band relative to the WT is shown below. Graphs represent mean of independent experiments +/- SD; n.s. = *p* ≥ 0.05 **(D)** GOF[OM] mutations do not disrupt RcsF/OmpA crosslinking. Immunoblot analysis of *in vivo* formaldehyde crosslinked samples probed with α-RcsF antibodies (left). Immunoblot quantification of RcsF/OmpA relative to the WT (right). Graphs represent mean of three independent experiments +/- SD. **(E)** GOF[OM] variants were treated with 0.75 μg/ml PMB for 40 min. Graphs represent mean β-galactosidase activity normalized to OD600, +/- SD. Significance throughout this figure is indicated in comparison to the WT as follows: n.s. = *p* ≥ 0.05, * = p < 0.01, ** = p < 0.01, *** = p < 0.001. **** = p < 0.0001. ND = not determined. For additional analysis of data (E) S4 Table.

We characterized five of the six most enriched variants (Fig 2B, orange). These five mutants showed significantly increased Rcs activity (Fig 4A). Among them, T132I gave the strongest phenotype, and the colonies were highly mucoid. We excluded RcsF(N78C) because introduction of an additional Cys residue resulted in abnormal intramolecular disulfide bonding of a large fraction of RcsF. We also rebuilt several additional mutants found to be enriched on lactose (S3 Table); these mutants displayed only a modest increase in Rcs activity, and we did not pursue them further. It is worth mentioning that since the complete derepression of Rcs is lethal, all of these mutants likely confer only a partial GOF phenotype.

We considered two mechanisms by which GOF[OM] mutants might increase signaling: first, they could prevent or alter RcsF/OMP interaction to expose signaling residues for interaction with IgaA, and second, they could increase RcsF affinity for IgaA independently of an OMP. To differentiate between the two possibilities, we examined the effect of these mutations at the IM (Fig 4B), since signaling would increase if the second was applicable. Importantly, under conditions of our expression system, the signal has not yet reached saturation, enabling such analysis (S8 Fig). When expressed from the pBAD18 plasmid, the IM variant of T132I was unstable regardless of arabinose concentration. The remaining four mutants did not show an increase in signaling compared with the WT (Fig 4B), confirming that their phenotype is OM-specific. As expected, these GOF[OM] mutants retained the ability to interact with IgaA at the IM (Fig 4C) and the OM (S9 Fig). We did not observe an increase in the levels of RcsF/IgaA compared with WT, further negating increased RcsF affinity for IgaA as a reason behind GOF phenotype.

GOF[OM] mutations targeted the same region, and in some cases, the same residues as found to be important for both OmpA and IgaA interaction (Fig 3 and S7B Fig). However, in contrast to the LOF[IM] mutants, GOF[OM] mutants retained the ability to assemble the RcsF/OmpA complex (Fig 4D), with only A55V being mildly affected with 50% decrease in RcsF/OmpA complex, which we confirmed using pull-down experiments (S10 Fig, see S11 Fig for direct comparison of A55 alleles). In summary, since all mutants formed the RcsF/OMP complex and signaling increased, we concluded that the complex adopted a different, more active conformation. This more active conformation is also a likely explanation behind increased signaling in A55V mutant despite a 50% reduction in RcsF/OMP complexes.

We also performed molecular dynamics simulations to test for possible effects of GOF[OM] substitutions on RcsF conformation based on the published crystal structure of RcsF CTD [13]. At the end of the simulations, there were no significant changes in the structure for any of the five mutant models (S12 Fig). These results further support the conclusion that conformational changes of the RcsF/OMP complex, rather than RcsF itself, are responsible for increased signaling.

To further test the conformational change hypothesis, we tested whether GOF[OM] variants can respond to the PMB treatment. We reasoned that if mutations favor RcsF/OMP conformation that mimics an activated state, GOF[OM] mutant would be signal blind. Indeed, we observed either no or only small change in Rcs activity upon PMB treatment, and in all cases fold change was significantly lower than in WT (Fig 4E and S4 Table).

### Characterization of RcsF variants defective in signaling from the OM

**Fig 5:**
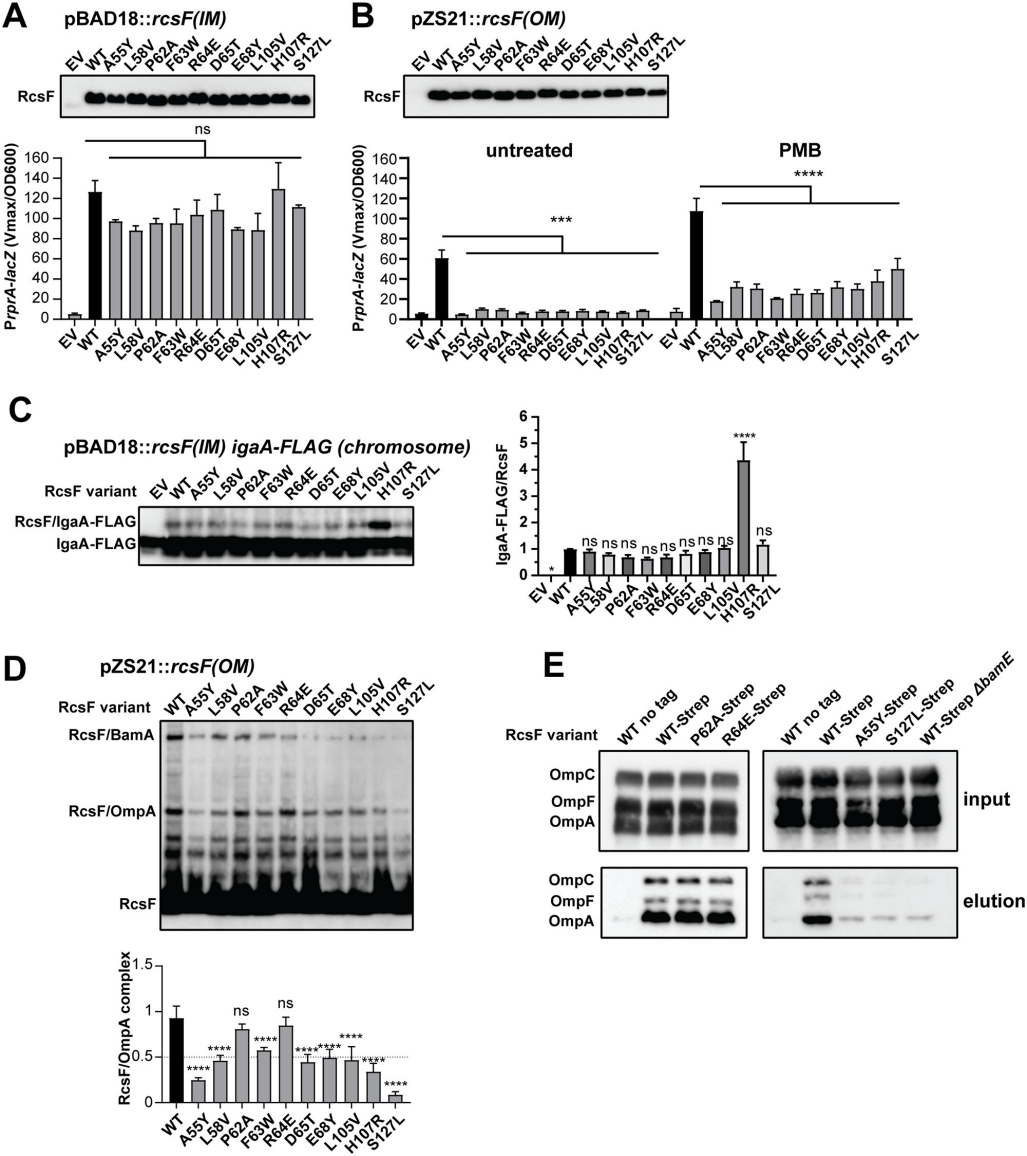
Characterization of RcsF LOF[OM] variants. LOF[OM] mutations were introduced on a plasmid encoding IM **(A)** or OM **(B)** *rcsF* and analyzed by immunoblotting with α-RcsF antibodies and by a β-galactosidase assay using a P*rprA-lacZ* transcriptional reporter. Where indicated, strains were treated with 0.75 μg/ml PMB for 40 min. Graphs represent mean β-galactosidase activity normalized to OD600, +/- SD. **(C)** LOF[OM] mutations were introduced into IM localized RcsF and tested for crosslinking to the chromosomally encoded IgaA-FLAG. Immunoblot analysis of *in vivo* formaldehyde crosslinked samples probed with α-FLAG. RcsF/IgaA band was quantified relative to the WT. Graph represents mean of independent experiments +/- SD. **(D)** LOF[OM] variants display varying levels of RcsF/OmpA crosslinking. Immunoblot analysis of *in vivo* formaldehyde crosslinked samples probed with α-RcsF antibodies (top). Immunoblot quantification of RcsF/OmpA relative to the WT (bottom). Graphs represent mean of three independent experiments +/- SD. **(E)** *In vivo* pull-down in the absence of crosslinking. Solubilized membrane fractions of strains expressing indicated RcsF variants were subjected to Streptactin sepharose purification. Immunoblots of input and elution fractions were probed with α-OmpA and α-OmpC/F antibodies. Pull-down quantification is in S14 Fig. Significance throughout this figure is indicated in comparison to the WT as follows: n.s. = *p* ≥ 0.05, * = p < 0.01, ** = p < 0.01, *** = p < 0.001. **** = p < 0.0001. ND = not determined. For additional significance comparison of data (A) and (B) see S6 Table.

Many more mutants were found to be depleted during growth on lactose, and they were indistinguishable from the nonsense *rcsF* mutants (Fig 2C). As expected, these included mutants defective in signaling to IgaA identified from the LOF[IM] screen (Fig 2C). To identify mutants specifically defective in signaling from the OM (S2 Fig), we focused on the non-proline mutant variants that did not display suppressor phenotype in the LOF[IM] screen (Fig 2 and S5 Table). We remade 18 candidate mutants (S5 Table), and selected ten for further analysis based on the following criteria: i) mutations did not affect RcsF protein levels when expressed at the IM or OM (Fig 5A and 5B); ii) mutations did not affect RcsF signaling to IgaA at the IM (Fig 5A).

All of the mutants displayed reduced signaling from the OM during normal growth conditions and upon PMB treatment, demonstrating the OM-specific signaling defect (Fig 5B). Next, we tested for the ability of RcsF to interact with IgaA at the IM (Fig 5C). One of the variants, RcsF(H107R), displayed increased RcsF/IgaA crosslinking (Fig 5C). The significance of this finding is currently not clear, since there was no increased in signaling activity of RcsF(H107R) at the IM (Fig 5A). Since formaldehyde targets amine and amide groups for crosslinking, it is possible that arginine substitution simply increases crosslinking efficiency by providing additional functional groups. Importantly for the scope of this study, none of the mutants disrupted the ability of RcsF to interact with IgaA at the IM, providing further evidence that these variants specifically cannot signal to IgaA from the OM. Consistent with this hypothesis, several of these mutants displayed a reduction in RcsF/IgaA crosslinking when expressed from the OM (S13 Fig).

All mutant residues mapped to the same region of RcsF and, in some cases, the same residues as before (Figs 3 and 4 and S7A–S7C Fig). When we performed crosslinking experiments, we observed several distinct of phenotypes. RcsF(P62A) and RcsF(R64E) accumulated WT levels of the RcsF/OmpA complexes (Fig 5D). Conversely, some mutants resulted in a reduction in RcsF/OmpA complexes to varying degrees, with the previously reported A55Y allele [8] and the novel S127L allele being the most affected (Fig 5D).

To confirm the RcsF/OMP assembly phenotype using an independent approach and to test for RcsF interaction with the remaining OmpC and OmpF partners, we performed a pull-down assay with an RcsF-Strep-tag from detergent-solubilized membrane fractions (Fig 5E). OmpA, OmpC, and OmpF stably interact with RcsF even without crosslinking and could be readily detected in elution fractions after co-purification with the WT RcsF-Strep. P62A and R64E variants behaved like the WT (Fig 5E and S14 Fig). Because the levels of RcsF/OMP complexes did not change, but the ability to signal was inhibited, we concluded that P62A and R64E substitutions likely favor the complex in the constitutively “OFF” conformation.

On the other hand, levels of OmpA, as well as OmpC and OmpF were dramatically decreased in the elution fractions of A55Y and S127L RcsF-Strep variants (Fig 5E and S14 Fig), confirming an assembly-defective phenotype. The importance of the A55 and S127 residues is supported by two independent screens, since the LOF[IM] A55K and S127K variants also abolished RcsF/OMP assembly (Fig 3, see S11 Fig for direct comparison of all A55 alleles). The assembly-defective phenotype of A55Y and S127L mutants is also very similar to the phenotype of the *ΔbamE* mutant, in which the Bam complex is unable to assemble RcsF/OMP complexes (Fig 5E and S14 Fig) [8,22,27]. However, in contrast to RcsF in the *ΔbamE* mutant [23,27], RcsF(A55Y) and RcsF(S127L) variants did not increase crosslinking to BamA (Fig 5D) and were likely retained in a periplasmic-exposed orientation.

Unlike A55K and S127K, A55Y and S127L retain their ability to signal to and interact with IgaA at the IM (Fig 5A and 5C). However, they could not signal properly from the OM (Fig 5B), demonstrating that when RcsF is tethered to the OM, its periplasmic localization in the absence of the OMP interaction is not sufficient to induce signaling, suggesting an active role for OMPs in regulating RcsF signal transduction.

## Discussion

In this study, we performed three independent genetic screens yielding distinct classes of *rcsF* signaling mutants (S2 Fig). These screens were unbiased and not narrowed by a specific hypothesis, as the site-saturated library covered the entire CTD rather than particular sites, and selection conditions were neutral to changes in RcsF/OMP interactions. Therefore, it is remarkable that all three screens identified the same region and often the same residues of RcsF CTD that are critically important for signal transduction. The screens also demonstrate that this region is bifunctional and mediates interaction with both IgaA and OMPs. Importantly, these two functions can be genetically separated, since we identified mutants that prevent RcsF/OMP formation without affecting the ability of RcsF to signal to IgaA.

**Fig 6:**
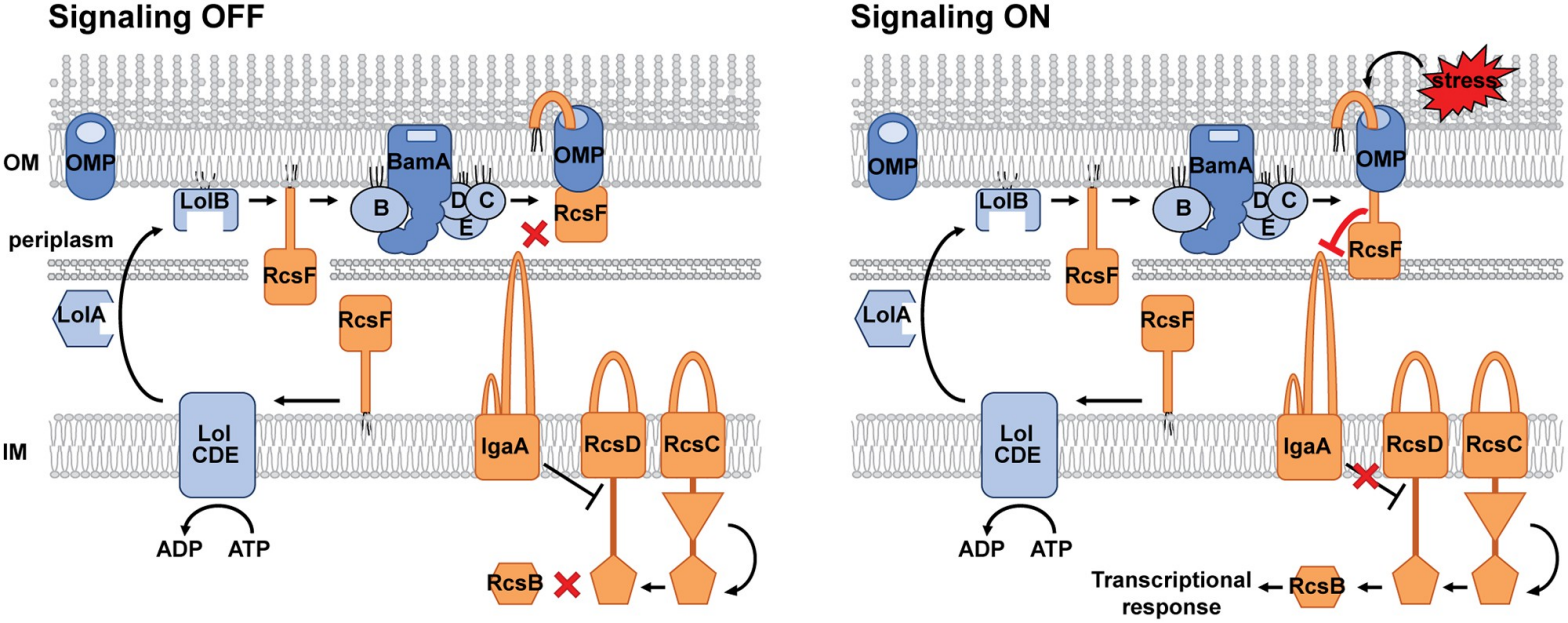
Proposed model for the mechanism of signal transduction from RcsF to IgaA. Under normal growth conditions, RcsF signaling residues important for interaction with IgaA are seated inside the lumen of the OM, keeping the signaling OFF. When OM stress is detected, the RcsF/OMP complex undergoes conformational changes that expose the signaling residues toward periplasm, allowing interaction with IgaA, thereby activating signaling.

The bifunctional nature of this region suggests that signaling involves a partner switch. Under unstressed conditions, this region of RcsF interacts with OMPs, while during stress conditions (or when RcsF is mislocalized to the IM), it mediates interaction with IgaA, indicating that these residues somehow become exposed for IgaA interaction. Both GOF[OM] and LOF[OM] screens provide genetic evidence for conformational changes in the RcsF/OMP complex rather than prevention of RcsF/OMP complex formation promoting signaling to IgaA (Fig 6). The GOF[OM] screen for constitutively signaling mutants did not yield any assembly-defective mutants that prevent RcsF/OMP complex formation; instead, all mutants formed RcsF/OMP complexes. By contrast, such assembly-defective mutants were identified in the LOF[OM] screen and could not properly signal from the OM. Like RcsF/OMP assembly defective mutants of the Bam complex [22], the phenotype of the RcsF(A55Y) and RcsF(S127L) variants supports the conclusion that RcsF/OMP is required for signaling and that retention of RcsF on the periplasmic side of the OM is not sufficient to induce signaling. However, if all that is needed to activate signaling is to make the RcsF region of the CTD accessible for IgaA interaction, why are these assembly-defective mutants not functional? We reason that the OMP plays a direct role in Rcs signaling by regulating the ability of RcsF to interact with IgaA from the OM. In this way, the cell will avoid unnecessary signaling mediated by RcsF transport intermediates in the absence of stress, which is both unnecessary and can be toxic to the cell [15,17]. Indeed, Rcs signaling in response to PMB was found to be independent of *de novo* protein synthesis [8], further supporting the idea that RcsF export intermediates are not the mediators of signal transduction.

How does the OMP stimulate ability of RcsF to signal to IgaA? Clearly, the OMP is not absolutely required for RcsF/IgaA interaction, because RcsF can signal in the absence of the OMP, for example, when mislocalized at the IM. One interesting possibility involves the potential role of the RcsF NTD. Deletion of the NTD in the context of mislocalized RcsF results in even higher Rcs activation [18], suggesting that the NTD may negatively regulate the ability of the CTD to signal. If so, the OMP could stimulate ability of RcsF to signal from the OM by sequestering the NTD even in the activated conformation (Fig 6).

We identified several mutants that favor the RcsF/OMP complex in a constitutive ON or OFF conformation. While more structural information about RcsF/OMP complexes is needed to fully understand the conformational changes, the simplest model involves “sliding” of important signaling residues toward the periplasm for interaction with IgaA. In this respect, it is interesting that this region together with the NTD is disordered and overall very similar to the disordered regions of colicins, protein toxins that slide inside the cell through the lumen of OMPs, in some cases though OmpC and OmpF [28–31].

Our new, refined model for how RcsF signals to IgaA (Fig 6) combines two somewhat opposing early ideas for the Rcs stress response [6,8,21]. On the one hand, the RcsF/OMP complex is a signaling complex, and its assembly is required for Rcs function. On the other hand, the OMP indeed occludes RcsF CTD from interacting with and signaling to IgaA under unstressed conditions. However, the OMP actually plays an active regulatory role, and modulation of RcsF/OMP interaction involves intricate conformational changes rather than inhibition of complex formation to enable RcsF interaction with IgaA. This new signaling model predicts the formation of a tripartite complex in which RcsF is bound to OMP and IgaA simultaneously. However, low expression levels of IgaA combined with a low fraction of RcsF that engages in signaling [6,23] make it difficult to capture this complex using in vivo crosslinking. Further studies are needed to characterize this complex biochemically. Likewise, it would be interesting to see whether mutations that alter the regulatory role of OMPs can be isolated. Thus far, genetic analysis of OMP function has been challenging because of their redundant role in RcsF pathway [6,21] while simultaneously having separate functions of their own and collectively being essential for the OM structural integrity [32–34].

One of the advantages of saturated screening is that it provides a quantitative phenotype of all mutants. While we characterized one representative mutant per codon, pairing them with remaining amino acid variant hits can help establish certain trends, predict the nature of protein interactions, and possibly explain some phenotypes. For example, one interesting feature of the signaling region in RcsF is the presence of many non-polar amino acids. All mutant hits that disrupted interactions with IgaA increased the charge in this region, while GOF[OM] and LOF[OM] mutants that retain the ability of RcsF to interact with IgaA at the IM largely preserved the hydrophobicity of these residues/region. This suggests that RcsF/IgaA interaction is driven, at least in part, by hydrophobic interactions, and the ColabFold structural predictions also support this idea. On the other hand, GOF[OM] and LOF[OM] substitution involved amino acids with longer side chains, in some cases bulkier hydrophobic amino acids. Considering that this region is normally seated within the relatively small polar lumen of the OMP, increasing the hydrophobicity and size of a side chain would not be favorable for this interaction. It is, therefore, not surprising that such substitutions either affected RcsF/OMP assembly or resulted in distinct RcsF/OMP conformations. In the case of GOF[OM], these hydrophobic residues could be pushed away from the polar lumen towards the periplasm, where they would likely be readily captured by the periplasmic domain of IgaA, given its high affinity for RcsF [23].

The periplasmic domain of IgaA is large, with more than 550 amino acids, and is predicted to span a substantial part of the periplasm. While there is no experimentally determined structure of IgaA and no detailed studies of what residues interact with RcsF, it is interesting that structural predictions of the RcsF/IgaA complex identified the RcsF binding site at the tip of the IgaA periplasmic domain. It suggests that RcsF may not need to traverse an entire periplasmic space to activate signaling. We are only beginning to understand how periplasmic width dictates the structure/function of trans-envelope protein complexes, but, intriguingly, the introduction of an additional unstructured linker between the NTD and the CTD of RcsF can compensate for the increased width of the periplasmic space [20].

The Rcs stress response is a complex signaling pathway that can detect and transmit the stress signal from the cell surface across the OM, periplasm, and IM to regulate gene expression [4]. While structural information is not available for most of the Rcs pathway components, our analysis of interactions between RcsF and OMPs or IgaA, paired with analysis of IgaA/RcsD interaction [35], illuminates phosphorylation-independent steps of signal transduction across layers of the cell envelope. This signal transduction essentially relies on a domino effect of conformational changes, in which envelope stress changes the conformation of the RcsF/OMP complex enabling RcsF to interact with the periplasmic domain of IgaA. This, in turn, changes how IgaA interacts with RcsD, allowing the phosphotransferase to proceed to activate the transcriptional factor RcsB. Interestingly, the periplasmic domain of IgaA is important for IgaA/RcsD interaction [35], suggesting that RcsF binding may directly modulate this interaction. Determining which molecular signal initiates conformational changes of the RcsF/OMP complex and how the Rcs system resets after the envelope damage is mitigated are exciting avenues for future research.

## Materials and methods

### Growth conditions

Unless otherwise stated, strains were grown in lysogeny broth (LB) (10 g/L tryptone, 5 g/L yeast extract, 10 g/L NaCl) at 37°C. Antibiotics were added when appropriate at the following concentrations: kanamycin (Kan) 25 μg/mL, ampicillin (Amp) 125 μg/mL. LB was supplemented with indicated amount of arabinose when applicable. All strains and primers used in this study are listed in S7 and S8 Tables. General strain construction is described in the S1 Materials and Methods.

### Genetic screening

Construction of the *rcsF* mutant library has been described in detail previously [22]. For LOF[IM] screen, plasmids pools of the individual codon libraries were independently transformed into the host strain MG2201 (*ΔlolB*, *Δlpp*, *ΔrcsF*, *pBAD18*::*lolB*) [17] and recovered on selective LB Kan 0.2% arabinose plates. For the selection, 10 mL LB Kan media with or without 0.2% arabinose was inoculated with pooled libraries at 10^5^ cells/mL (for approximately 11 generation selection), and cultures were grown in flasks at 37°C with orbital shaking until reached saturation, approximately 18 hours. For GOF[OM] and LOF[OM] screens, plasmid pools of the individual codon libraries were independently transformed into the host strain AK-266 (*ΔrcsF PrprA-lacZYA)* [8] and recovered on selective LB Kan plates. For selection, 10 mL M9 minimal media (26.1 mM Na2HPO4, 22 mM KH2PO4, 8.5 mM NaCl, 18.6 mM NH4Cl, 1 mM MgSO4, 100 μg/mL thiamine, with 0.2% lactose or 0.2% glucose as indicated) was inoculated with pooled libraries at 10^5^ cells/mL for GOF[OM] screen (approximately 11 generations selection), or at 10^7^ cells/mL for LOF[OM] screen (approximately 6 generation selection), and cultures were grown in flasks at 37°C with orbital shaking until reached saturation, approximately 13 hours.

All selections were carried out in triplicate. After that, cells were collected by centrifugation, plasmids were isolated, and prepared for amplicon GENEWIZ sequencing and analysis as described in detail [22]. All data for individual variants and log2[FC] calculations are presented in S1 Dataset. GraphPad Prism 9.0 was used to generate graphs and for statistical analysis. All mutant hits were rebuilt *de novo*, transformed into a clean genetic background, and phenotypically reconfirmed before further analysis.

### β-galactosidase assay

For strains with pZS21::*rcsF*(OM) variants, overnight cultures were diluted 1:100 and grown in LB Kan for 1.5 h until OD_600_ 0.5 was reached. PMB was added to a final concentration of 0.75 μg/mL, and strains were grown for an additional 40 min. For pBAD18::*rcsF*(IM) variants, overnight cultures were diluted 1:500 and grown in LB Amp with 2*10^−4^% arabinose for 3 h to allow expression of *rcsF*. 100 μL of each sample was taken for β-galactosidase assay, as described previously [8]. Vmax, determined using Gen5 software (BioTek), was normalized to OD_600_. Experiments were performed in at least three biological replicates, and mean values +/- SD were plotted. GraphPad Prism 9.0 was used to generate graphs for statistical analysis.

### *In vivo* crosslinking and immunoblot analysis

*In vivo* formaldehyde crosslinking and immunoblot analysis was performed on cells from the exponentially grown cultures as described [27]. Immunoblots for determination of total RcsF levels were taken from the same cultures used for β-galactosidase assay as described above. Immunoblots were visualized and quantified using the ChemiDoc MP Imaging System (Bio-Rad). Intensity of crosslinking bands was normalized by the total signal of corresponding protein (RcsF or IgaA-FLAG), presented as fold change of the WT. GraphPad Prism 9.0 was used to generate graphs for statistical analysis. All figures are representative images from at least three independent biological replicates.

### *In vivo* pull-down assay with *rcsF* variants

Corresponding plasmids (pZS21::*rcsF*, pZS21::*rcsF*-Strep, and its mutant derivatives) were transformed into AK-266 or AK-688. Strains harboring the plasmids were grown till mid-log in 50 mL culture at 37°C, after which cells were harvested by centrifugation. Cell pellets were resuspended in 5 mL of buffer A (25 mM Tris–HCl pH 8.0, 150 mM NaCl, 0.1mM PMSF (Sigma-Aldrich), and 1X Protease Inhibitor Cocktail (Thermo Scientific), 0.2 mg/mL lysozyme (Gold Biotechnology)) and disrupted by Emulsi Flex (Avestin) with 30 psi pressure. Cell lysates were clarified by centrifugation, after which membrane fractions were isolated by ultracentrifugation (XL-100K, Beckman Coulter) at 100,000 g for 60 mins at 4°C. The membrane pellets were solubilized in 2 mL of buffer B (25 mM Tris–HCl pH 8.0, 150 mM NaCl, 1% DDM (Gold Biotechnology)) overnight at 4°C with mild agitation. After solubilization, the protein concentration was determined using the BCA protein assay kit (Thermo Scientific). Equal amount of proteins (400 μg/mL) of each strain was used as an input and applied to pre-equilibrated columns (Thermo Scientific) containing 200 μL of Streptactin Sepharose (IBA Life sciences). Columns were washed 10 times with 500 μL of buffer C (25 mM Tris–HCl pH 8.0, 150 mM NaCl, and 0.02% DDM) and eluted with 200 μL buffer C containing 5mM deshthiobiotin (Sigma-Aldrich). Equal amounts of input and elution fractions from each strain were analyzed by immunoblotting. Experiments were performed in three independent biological replicates.

## Supporting information

S1 Fig Proposed model of RcsF/OMP complexes using OmpC and OmpF partners. The models are based on topology studies [21], OMP sites of crosslinked RcsF G60-pBPA [21], as well as overall site-specific crosslinking patterns [6,21,23]. OMPs are colored in green; only the monomer is shown for simplicity. RcsF is colored in magenta, Cys16 (+1 residue) with its lipid moieties are colored in red. RcsF residues shown to crosslink to OMP in various studies are represented as cyan spheres and listed on the right. * indicates residues mutations of which do not affect RcsF/OMP interaction [22]. RcsF residues 53–65 (junction region between NTD and the folded core domain) are predicted to span the lumen of OMPs, which additionally occludes several core domain residues. RcsF residues 31–53 are predicted to crosslink to extracellular loops of OMPs. Note: OmpA contains a C-terminal periplasmic domain, which may account for some crosslinks within RcsF folded core. Complexes are modeled based on RcsF (PDB 2Y1B), OmpC (PDB 2J1N) or OmpF (PDB 2OMF).
(TIF)

S2 Fig Genetic strategy to isolate distinct classes of *rcsF* signaling mutants. Representative mutant variants belonging to each class are indicated.
(TIF)

S3 Fig Experimental setup to study RcsF(IM) and its variants. (A). Prolonged expression of RcsF(IM) results in growth inhibition. Strains carrying indicated pBAD18 plasmids with the WT RcsF(OM) or signal sequence mutant causing retention at the IM, RcsF(IM), were grown overnight in the absence of arabinose and diluted 1:500 in LB supplemented with 2*10^−4^% arabinose where indicated. Arrow indicates the time point that was used for all experiments in this study. After 3 hrs, cultures were diluted to monitor further growth. (B) Comparison of RcsF protein levels (top) and Rcs activity (bottom) in strains with different *rcsF* expression vectors. Rcs activity was measured by β-galactosidase assay using P*rprA-lacZ* transcriptional reporter. Graphs represent mean β-galactosidase activity normalized to OD600 +/- SEM. Statistical analysis was performed comparing to the pZS21::*rcsF(OM)*: n.s. = *p* ≥ 0.05, **** = p < 0.0001.
(TIF)

S4 Fig IgaA-FLAG is fully functional. ***(A)*** P1 cotransduction frequency between *igaA*::*kan malT*::*Tn10* was quantified by % of KanR transductants out of the total number of TetR transductants (based on 100 colonies). As expected, *igaA* is essential in *att*Tn7::EV background, resulting in the linkage disruption. *igaA* is no longer essential in the *att*Tn7::*igaA-*FLAG background, with the cotransduction frequency comparable to *ΔrcsB*. ***(B)*** *att*Tn7::*igaA-*FLAG fully complements Rcs signaling under both stressed and unstressed conditions based on the β-galactosidase assay using P*rprA-lacZ* transcriptional reporter. Strains were treated with 0.75 μg/ml PMB for 40 min. Graphs represent mean β-galactosidase activity normalized to OD600 +/- SD.
(TIF)

S5 Fig LOF[IM] mutations disrupt crosslinking between RcsF and plasmid-encoded IgaA-FLAG. Immunoblot analysis of *in vivo* formaldehyde crosslinked samples and the validation of the RcsF/IgaA crosslinking band. The membrane was probed with mouse α-FLAG and rabbit α-RcsF antibodies. To facilitate simultaneous detection, the membrane was probed with Bio-Rad Anti-Mouse IgG StarBright Blue 700 and Anti-Rabbit IgG StarBright Blue 520 secondary antibodies. Membranes were visualized using the ChemiDoc MP Imaging System (Bio-Rad). Top panels are the black-and-white images of the single-channel fluorescent images; the bottom panel is the colored overlay of the single-channel images. The immunoblot quantification is not presented since no RcsF/IgaA bands were detected in LOF[IM] mutant samples.
(TIF)

S6 Fig RcsF/IgaA complex structure prediction using ColabFold. Amino acid sequences of the RcsF (green) devoid its signal sequence and the lipid-modified cysteine residue together with the full-length IgaA (cyan) were analyzed using Google Collab interface (see S1 Materials and Methods). The top-scoring structural model is shown as a complex overview (A) and the detailed RcsF/IgaA interface (B). The residues identified to mediate the RcsF/IgaA interaction based on the experimental analysis (LOF[IM] screen) are colored in red, and their sidechains are visualized.
(TIF)

S7 Fig Location of RcsF mutant residues identified from the different genetic screens in the context of the proposed RcsF/OMP model. OmpF is colored in gray; only a monomer is shown for simplicity. RcsF is colored in magenta, Cys16 (+1 residue) with its lipid moieties are colored in green. Residues are highlighted by colored spheres and were identified from the LOF[IM] screen (A), the GOF[OM] screen (B), and the LOF[OM] screen (C).
(TIF)

S8 Fig RcsF(IM) experimental setup enables the detection of increased Rcs activity. When RcsF(IM) expression is induced by a low concentration of arabinose (2*10^−4^%) used in this study, the Rcs activity has not yet reached saturation. Rcs activity can be further increased when the RcsF(IM) is overexpressed (o/e) by using 0.2% arabinose. Graphs represent mean β-galactosidase activity normalized to OD600 +/- SD.
(TIF)

S9 Fig GOF[OM] mutations do not affect crosslinking between RcsF and plasmid-encoded IgaA-FLAG. Immunoblot analysis of *in vivo* formaldehyde crosslinked samples probed with α-FLAG, and quantification of RcsF/IgaA-Flag band relative to the WT. Graphs represent the mean of independent experiments +/- SD; n.s. = *p* ≥ 0.05.
(TIF)

S10 Fig A55V but not T53F affect RcsF/OMP interaction. In vivo pull-down analysis in the absence of crosslinking. Solubilized membrane fractions of strains expressing indicated RcsF variants were subjected to Streptactin sepharose purification. Immunoblots of input and elution fractions were probed with α-OmpA and α-OmpC/F antibodies. Graphs represent the quantification of OMP bands relative to the WT; mean +/- SD; n.s. = *p* ≥ 0.05, ** = p < 0.01, **** = p < 0.0001.
(TIF)

S11 Fig Comparison of A55 mutant alleles. The graphs are based on the data presented in the Main Text compiled together for a side-by-side comparison. (A-C) Immunoblot quantification of *in vivo* formaldehyde crosslinked samples. (A) crosslinking between RcsF(IM) and chromosomal IgaA-FLAG. (B) crosslinking between RcsF(OM) and plasmid-encoded IgaA-FLAG. (C) Crosslinking between RcsF(OM) and OmpA. (D) In vivo pull-down analysis in the absence of crosslinking. Graphs represent the quantification of OmpA band relative to the WT; mean +/- SD. The representative immunoblot is on the right. A55 alleles compared to the assembly-defective *ΔbamE* mutant.
(TIF)

S12 Fig Molecular dynamics simulation results for the five RcsF GOF[OM] models. (A) Crystal structure of RcsF (PDB ID 2Y1B). Two non-consecutive disulfide bonds (Cys^74^−Cys^118^ and Cys^109^−Cys^124^) and the five single-point mutation residues are represented by sticks. (B) Overlaid views of the initial structure (red) and a 500-ns snapshot (blue). There are no distinct changes in the folded structure of the five models. (C) Averaged root-mean-square deviation (RMSD) values of the five models using their 500-ns trajectories show < 3 Å change with respect to the crystal structure. (D) RMSD time series throughout the 500-ns simulation for all five models.
(TIF)

S13 Fig LOF[OM] mutations lower crosslinking between RcsF and plasmid-encoded IgaA-FLAG. Immunoblot analysis of *in vivo* formaldehyde crosslinked samples probed with α-FLAG, and quantification of RcsF/IgaA-Flag band relative to the WT. Graphs represent mean of independent experiments +/- SD; n.s. = *p* ≥ 0.05, * = p < 0.01.
(TIF)

S14 Fig Quantification of OMP bands in the elution fraction after in vivo pull-down (related to the Fig 5D). Bands are quantified relative to the WT; mean of independent experiments+/- SD; n.s. = *p* ≥ 0.05, **** = p < 0.0001.
(TIF)

S1 Table Hits from LOF(IM) genetic screen. (DOCX)

S2 Table Statistical analysis for β-galactosidase assay data presented in Fig 3A and 3B. (DOCX)

S3 Table Hits from GOF(OM) the genetic screen. (DOCX)

S4 Table Statistical analysis for β-galactosidase assay data presented in Fig 4D. (DOCX)

S5 Table Hits from LOF(OM) genetic screen. These hits are non-Proline mutants and behaved like WT in the LOF(IM) screen.
(DOCX)

S6 Table Statistical analysis for β-galactosidase assay data presented in Fig 5A and 5B. (DOCX)

S7 Table Strains used in this study. Unless otherwise indicated, the host background is MC4100 (JCM158).
(DOCX)

S8 Table Primers used in this study. (DOCX)

S1 Materials and Methods Supplemental Materials and Methods. (DOCX)

S1 Dataset Performance of RcsF mutant library in three genetic screens. Data was collected and analyzed as described in Materials and Methods. “Reads (sum)” represents a sum of non-identical nucleotides reads encoding the same a.a. variant. Stop codon indicated by “*”. “Read fraction” represents a Read Sum normalized by a total nucleotide read count in that NGS sample. “Read fraction” was used for log2[FC] calculations. If the variant was not detected post-selection, we used relative abundance corresponding to 2 reads (below the threshold) to facilitate the log2[FC] calculation.
(XLSX)

S2 Dataset The underlying numerical data for all graphs (Figs 3–5) and the summary statistics. (XLSX)
