# Supplementary figures and images for: Conformational rearrangements in the sensory RcsF/OMP complex mediate signal transduction across the bacterial cell envelope
Source: PLoS Genet. 2023 Jan 27;19(1):e1010601. doi: 10.1371/journal.pgen.1010601 (PMC9907809; doi:10.1371/journal.pgen.1010601)

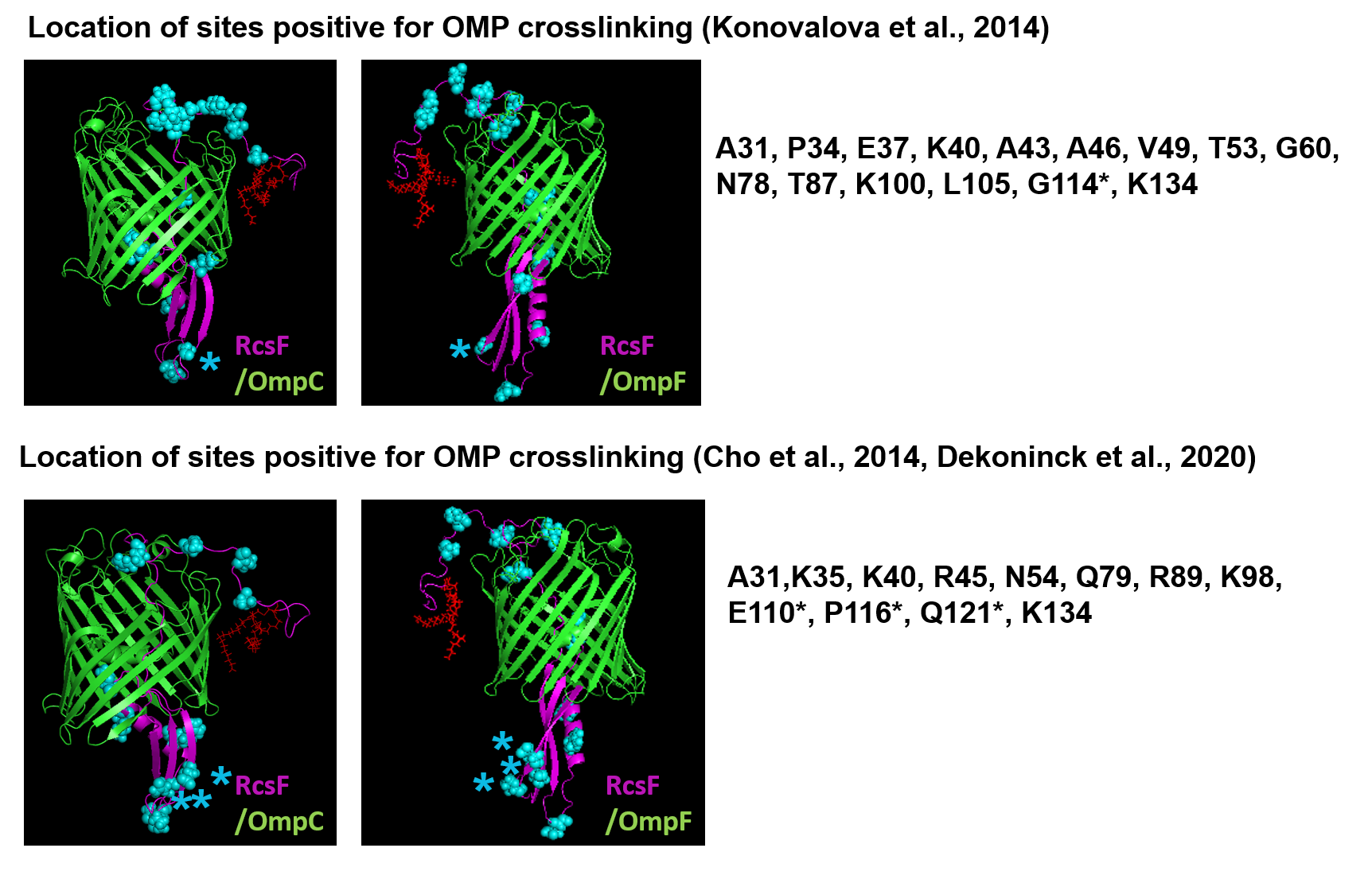

Supplement: S1 Fig — The models are based on topology studies [21], OMP sites of crosslinked RcsF G60-pBPA [21], as well as overall site-specific crosslinking patterns [6,21,23]. OMPs are colored in green; only the monomer is shown for simplicity. RcsF is colored in magenta, Cys16 (+1 residue) with its lipid moieties are colored in red. RcsF residues shown to crosslink to OMP in various studies are represented as cyan spheres and listed on the right. * indicates residues mutations of which do not affect RcsF/OMP interaction [22]. RcsF residues 53–65 (junction region between NTD and the folded core domain) are predicted to span the lumen of OMPs, which additionally occludes several core domain residues. RcsF residues 31–53 are predicted to crosslink to extracellular loops of OMPs. Note: OmpA contains a C-terminal periplasmic domain, which may account for some crosslinks within RcsF folded core. Complexes are modeled based on RcsF (PDB 2Y1B), OmpC (PDB 2J1N) or OmpF (PDB 2OMF). (TIF) [file pgen.1010601.s001.tif]

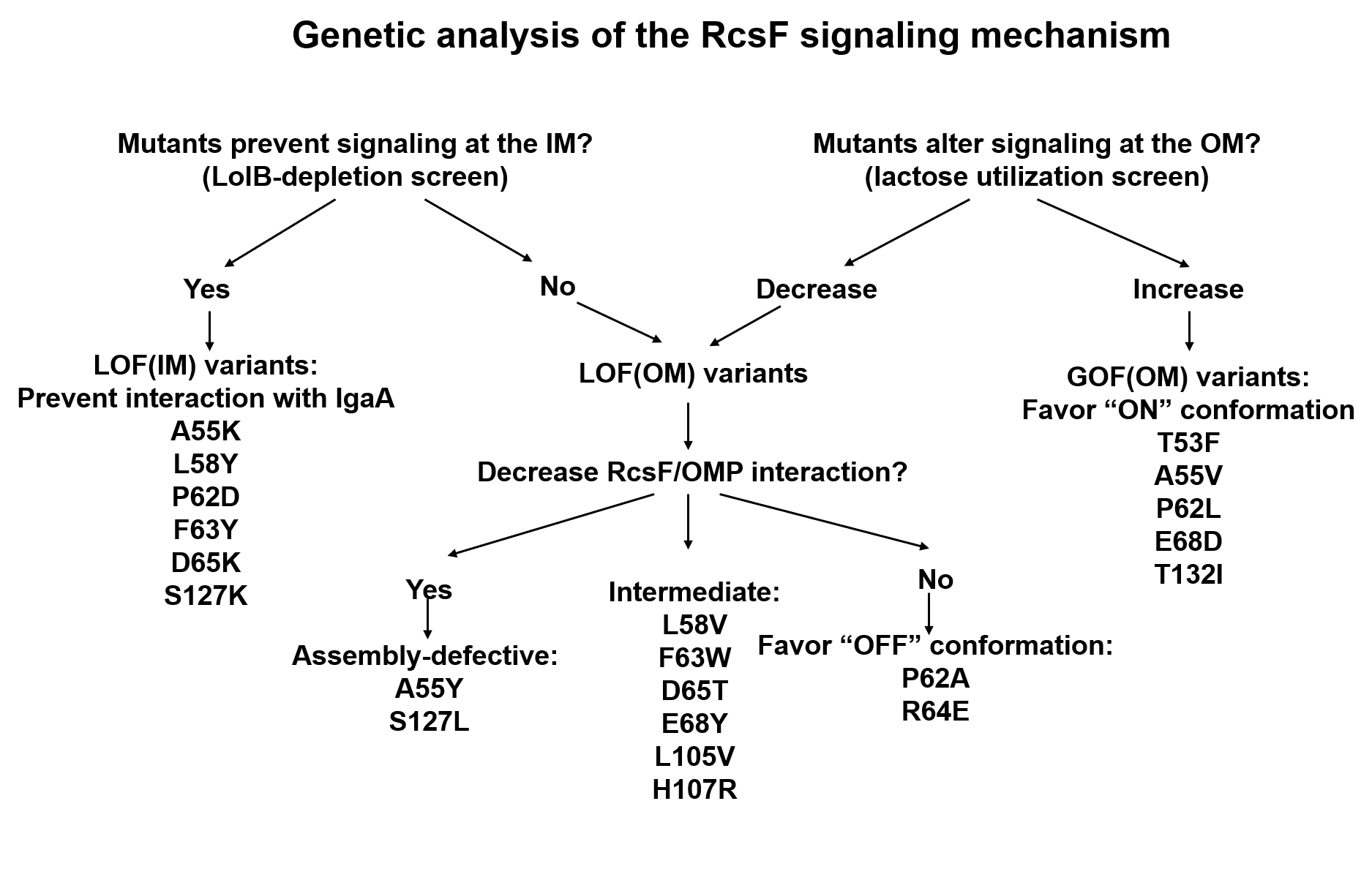

Supplement: S2 Fig — Representative mutant variants belonging to each class are indicated. (TIF) [file pgen.1010601.s002.tif]

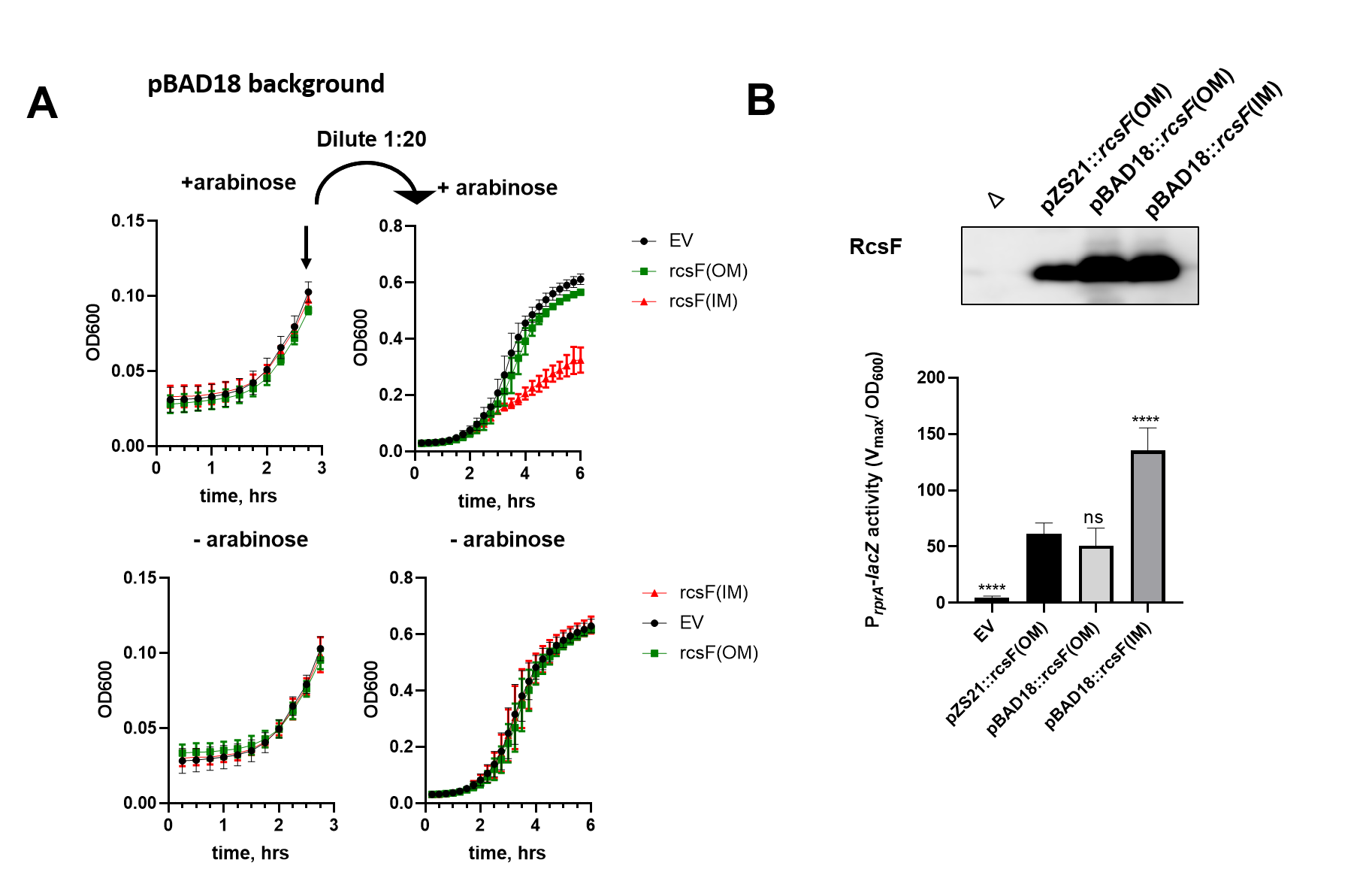

Supplement: S3 Fig — (A). Prolonged expression of RcsF(IM) results in growth inhibition. Strains carrying indicated pBAD18 plasmids with the WT RcsF(OM) or signal sequence mutant causing retention at the IM, RcsF(IM), were grown overnight in the absence of arabinose and diluted 1:500 in LB supplemented with 2*10−4% arabinose where indicated. Arrow indicates the time point that was used for all experiments in this study. After 3 hrs, cultures were diluted to monitor further growth. (B) Comparison of RcsF protein levels (top) and Rcs activity (bottom) in strains with different rcsF expression vectors. Rcs activity was measured by β-galactosidase assay using PrprA-lacZ transcriptional reporter. Graphs represent mean β-galactosidase activity normalized to OD600 +/- SEM. Statistical analysis was performed comparing to the pZS21::rcsF(OM): n.s. = p ≥ 0.05, **** = p < 0.0001. (TIF) [file pgen.1010601.s003.tif]

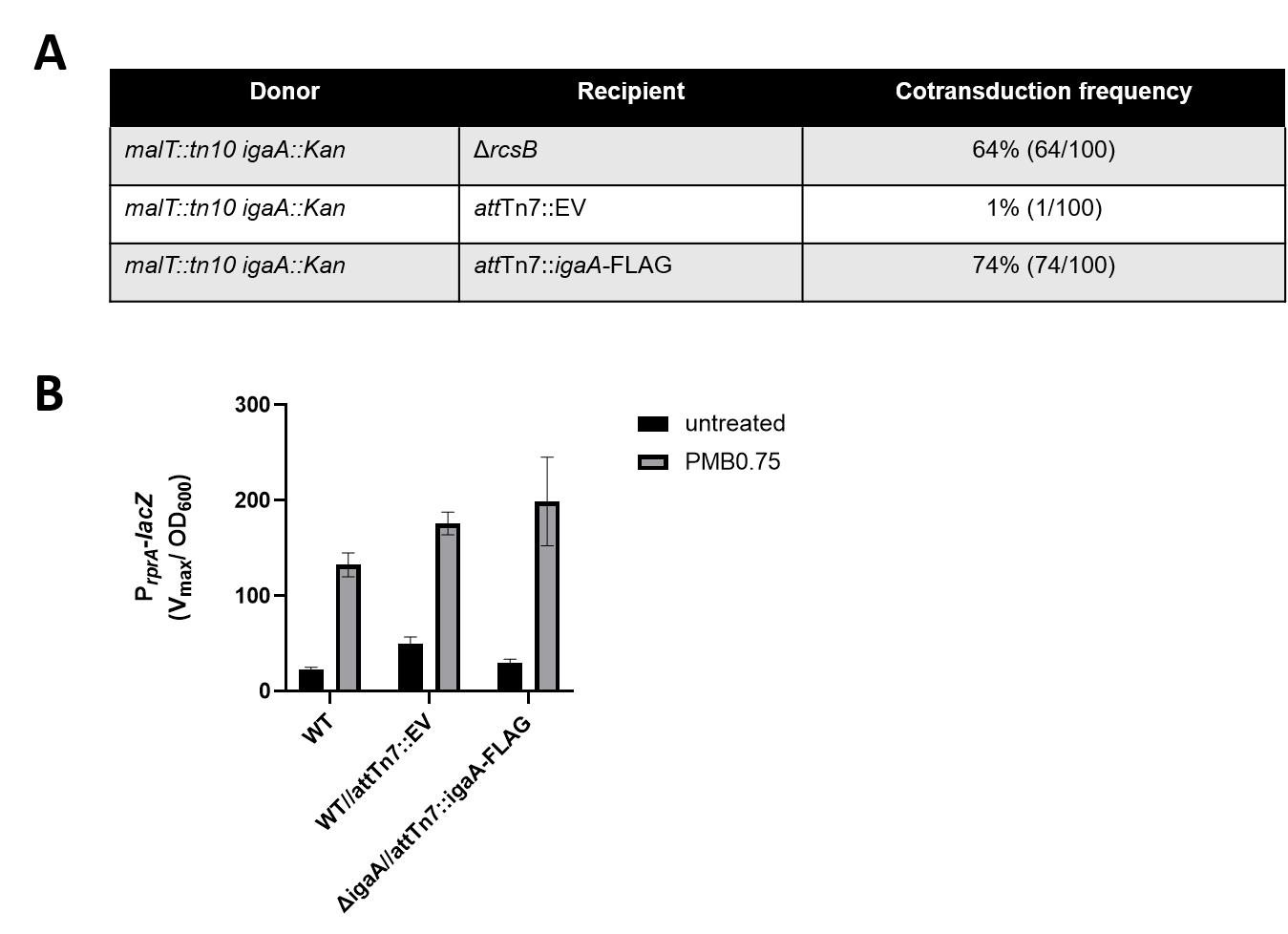

Supplement: S4 Fig — (A) P1 cotransduction frequency between igaA::kan malT::Tn10 was quantified by % of KanR transductants out of the total number of TetR transductants (based on 100 colonies). As expected, igaA is essential in attTn7::EV background, resulting in the linkage disruption. igaA is no longer essential in the attTn7::igaA-FLAG background, with the cotransduction frequency comparable to ΔrcsB. (B) attTn7::igaA-FLAG fully complements Rcs signaling under both stressed and unstressed conditions based on the β-galactosidase assay using PrprA-lacZ transcriptional reporter. Strains were treated with 0.75 μg/ml PMB for 40 min. Graphs represent mean β-galactosidase activity normalized to OD600 +/- SD. (TIF) [file pgen.1010601.s004.tif]

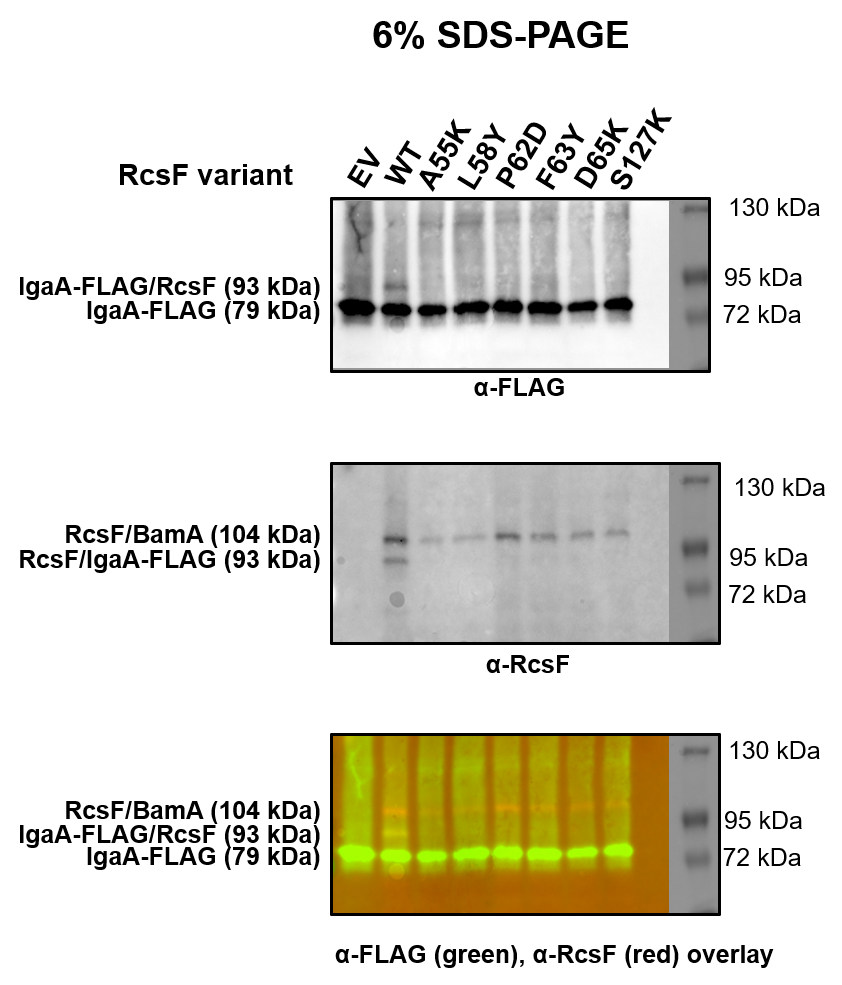

Supplement: S5 Fig — Immunoblot analysis of in vivo formaldehyde crosslinked samples and the validation of the RcsF/IgaA crosslinking band. The membrane was probed with mouse α-FLAG and rabbit α-RcsF antibodies. To facilitate simultaneous detection, the membrane was probed with Bio-Rad Anti-Mouse IgG StarBright Blue 700 and Anti-Rabbit IgG StarBright Blue 520 secondary antibodies. Membranes were visualized using the ChemiDoc MP Imaging System (Bio-Rad). Top panels are the black-and-white images of the single-channel fluorescent images; the bottom panel is the colored overlay of the single-channel images. The immunoblot quantification is not presented since no RcsF/IgaA bands were detected in LOF[IM] mutant samples. (TIF) [file pgen.1010601.s005.tif]

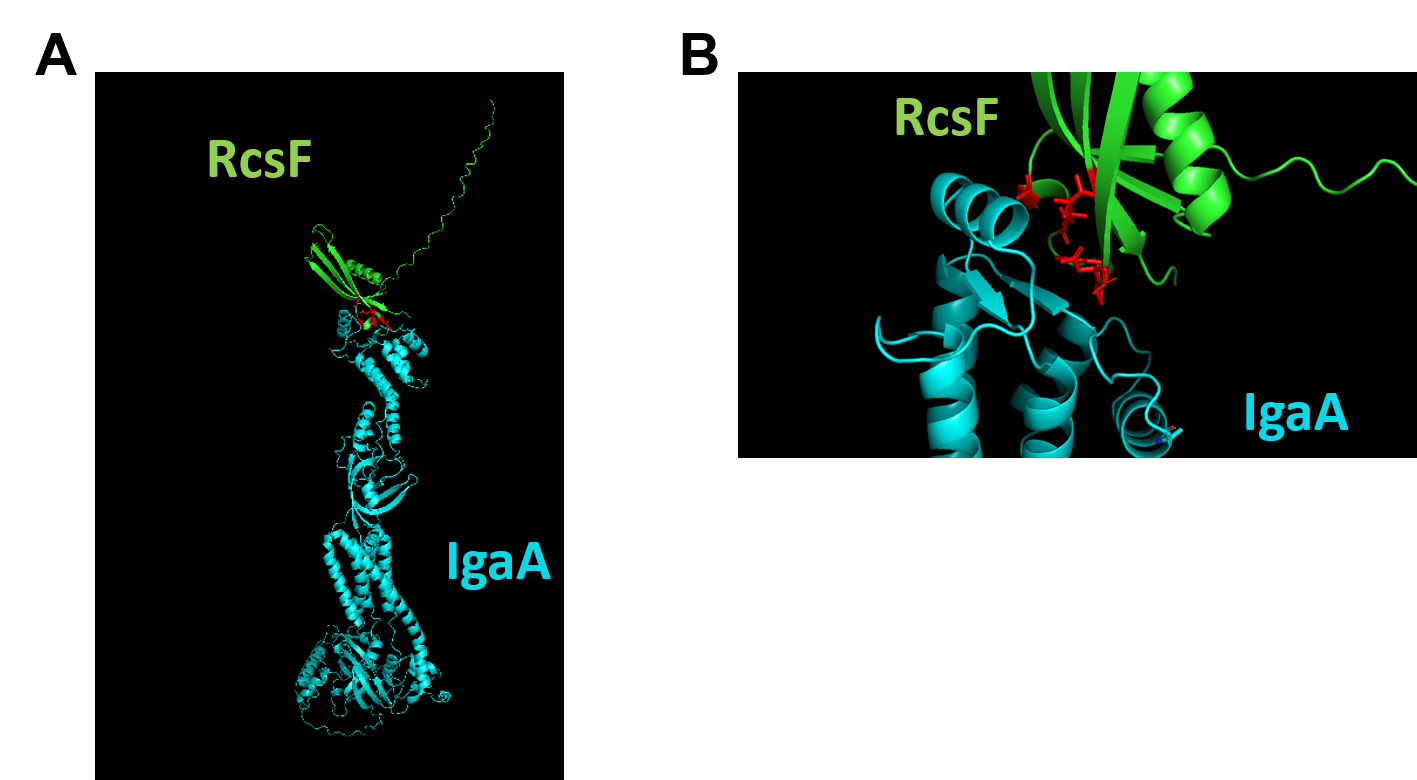

Supplement: S6 Fig — Amino acid sequences of the RcsF (green) devoid its signal sequence and the lipid-modified cysteine residue together with the full-length IgaA (cyan) were analyzed using Google Collab interface (see S1 Materials and Methods). The top-scoring structural model is shown as a complex overview (A) and the detailed RcsF/IgaA interface (B). The residues identified to mediate the RcsF/IgaA interaction based on the experimental analysis (LOF[IM] screen) are colored in red, and their sidechains are visualized. (TIF) [file pgen.1010601.s006.tif]

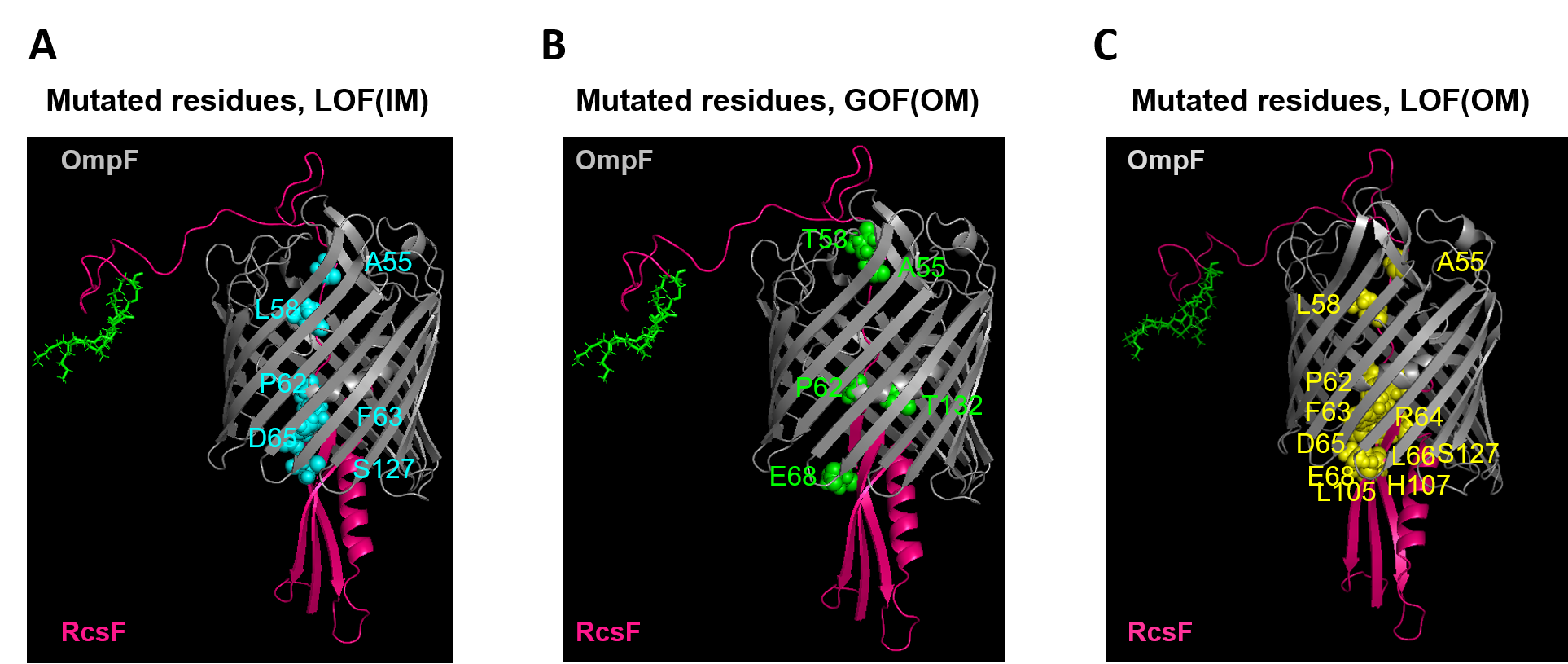

Supplement: S7 Fig — OmpF is colored in gray; only a monomer is shown for simplicity. RcsF is colored in magenta, Cys16 (+1 residue) with its lipid moieties are colored in green. Residues are highlighted by colored spheres and were identified from the LOF[IM] screen (A), the GOF[OM] screen (B), and the LOF[OM] screen (C). (TIF) [file pgen.1010601.s007.tif]

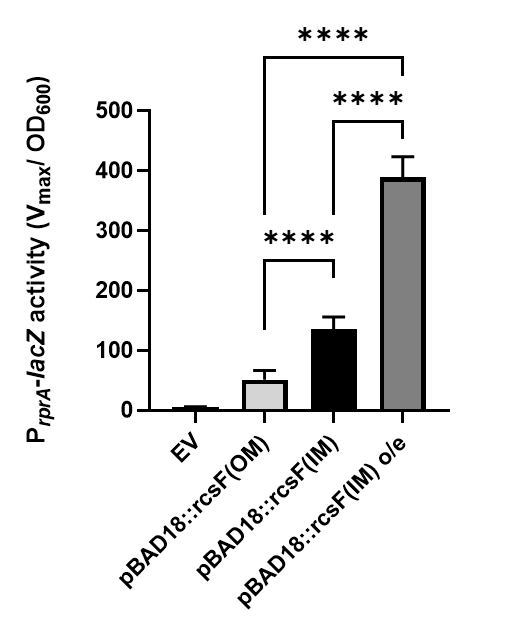

Supplement: S8 Fig — When RcsF(IM) expression is induced by a low concentration of arabinose (2*10−4%) used in this study, the Rcs activity has not yet reached saturation. Rcs activity can be further increased when the RcsF(IM) is overexpressed (o/e) by using 0.2% arabinose. Graphs represent mean β-galactosidase activity normalized to OD600 +/- SD. (TIF) [file pgen.1010601.s008.tif]

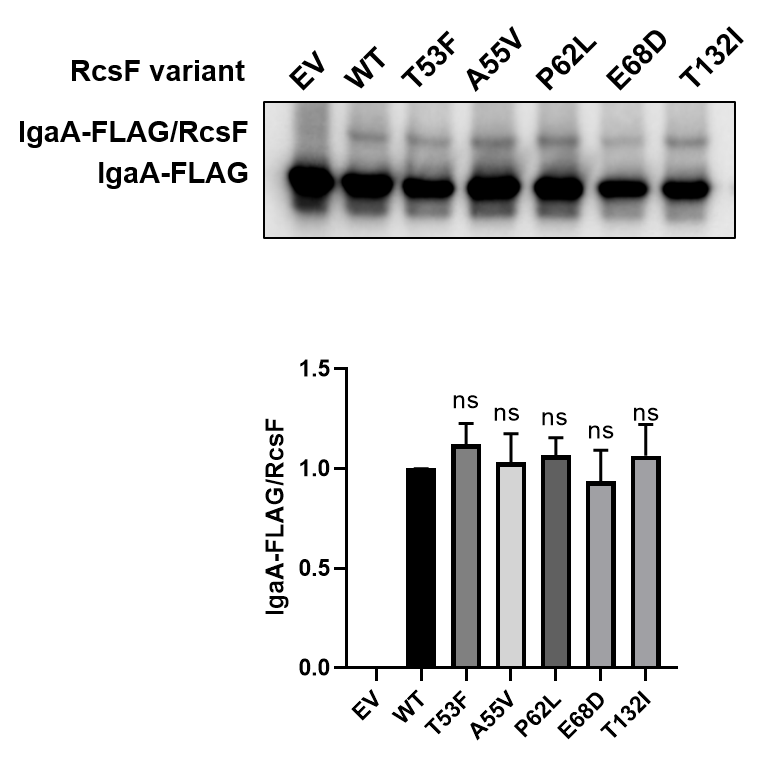

Supplement: S9 Fig — Immunoblot analysis of in vivo formaldehyde crosslinked samples probed with α-FLAG, and quantification of RcsF/IgaA-Flag band relative to the WT. Graphs represent the mean of independent experiments +/- SD; n.s. = p ≥ 0.05. (TIF) [file pgen.1010601.s009.tif]

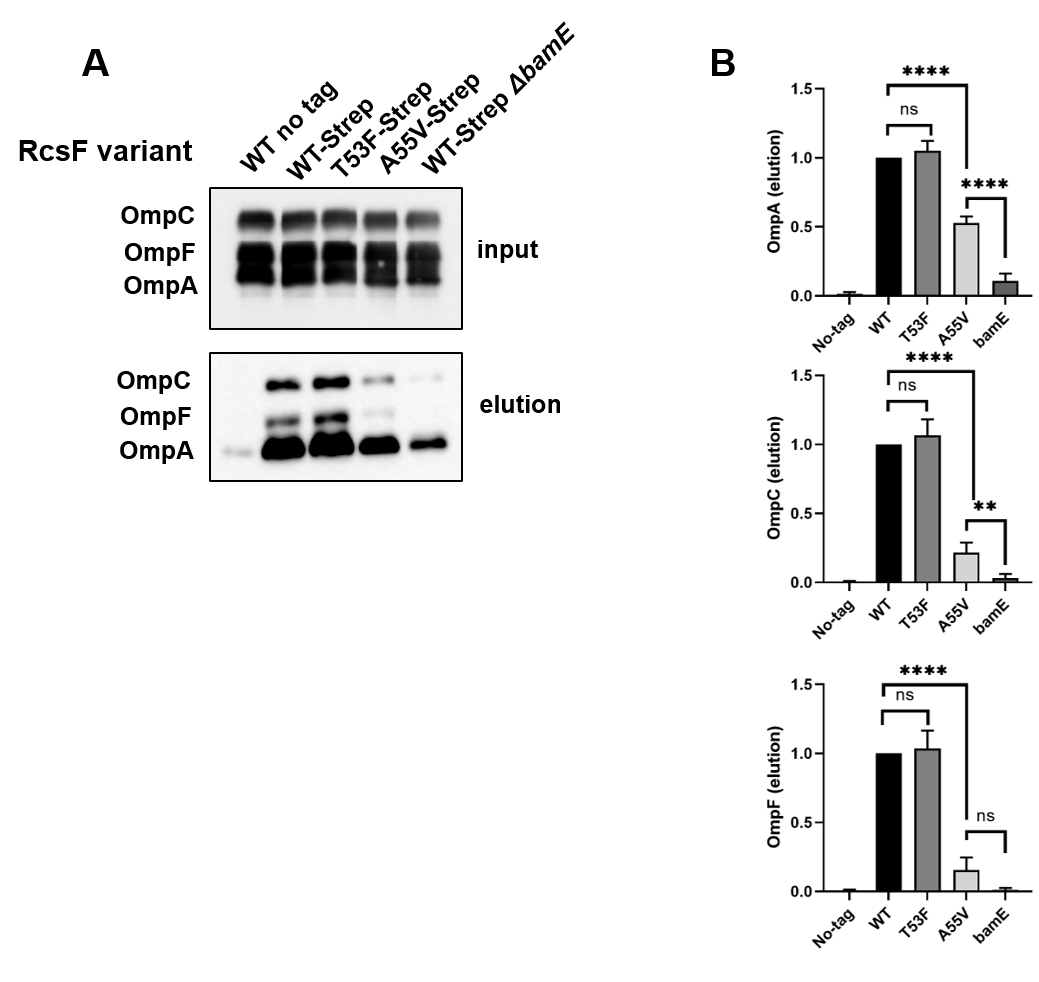

Supplement: S10 Fig — In vivo pull-down analysis in the absence of crosslinking. Solubilized membrane fractions of strains expressing indicated RcsF variants were subjected to Streptactin sepharose purification. Immunoblots of input and elution fractions were probed with α-OmpA and α-OmpC/F antibodies. Graphs represent the quantification of OMP bands relative to the WT; mean +/- SD; n.s. = p ≥ 0.05, ** = p < 0.01, **** = p < 0.0001. (TIF) [file pgen.1010601.s010.tif]

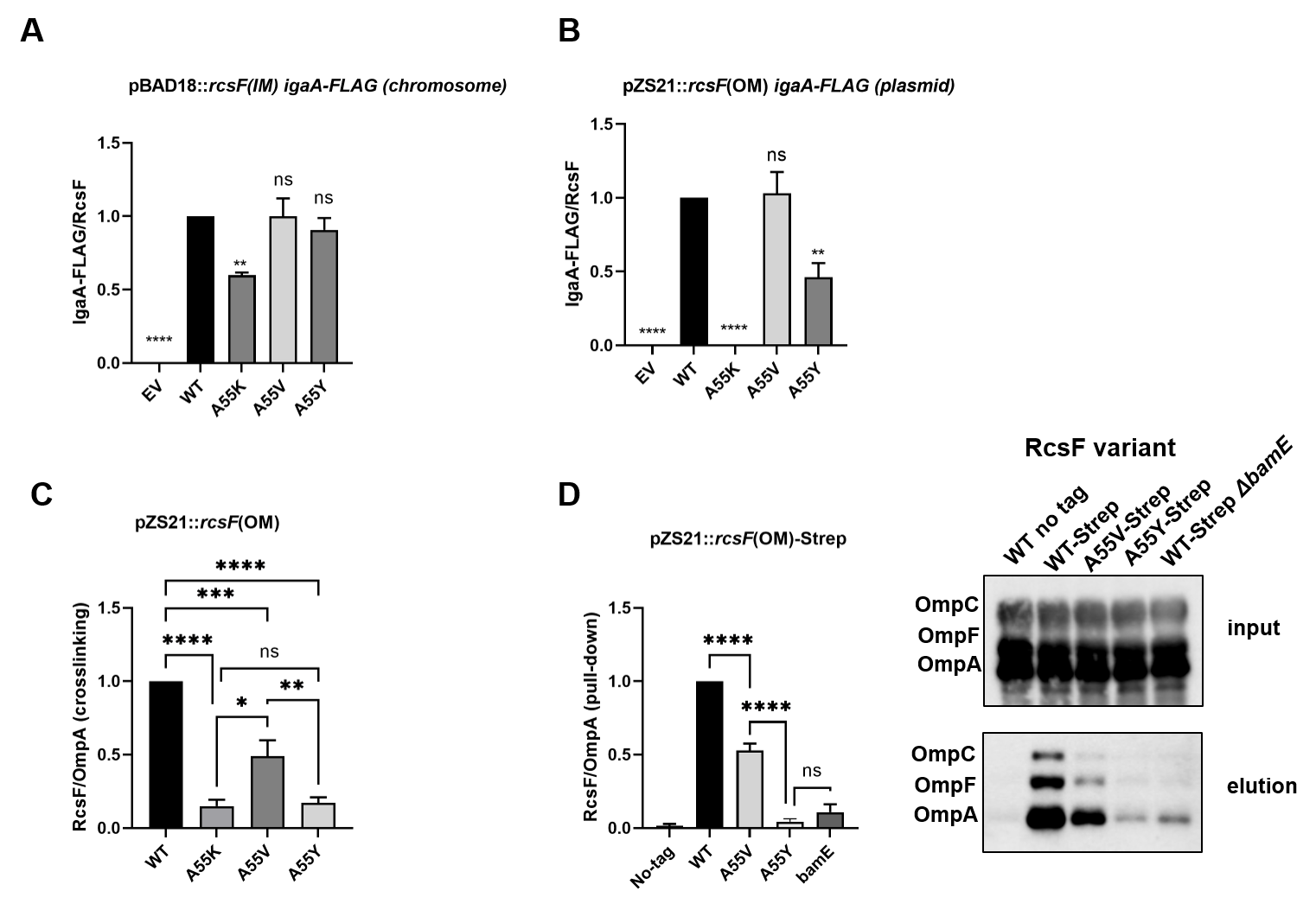

Supplement: S11 Fig — The graphs are based on the data presented in the Main Text compiled together for a side-by-side comparison. (A-C) Immunoblot quantification of in vivo formaldehyde crosslinked samples. (A) crosslinking between RcsF(IM) and chromosomal IgaA-FLAG. (B) crosslinking between RcsF(OM) and plasmid-encoded IgaA-FLAG. (C) Crosslinking between RcsF(OM) and OmpA. (D) In vivo pull-down analysis in the absence of crosslinking. Graphs represent the quantification of OmpA band relative to the WT; mean +/- SD. The representative immunoblot is on the right. A55 alleles compared to the assembly-defective ΔbamE mutant. (TIF) [file pgen.1010601.s011.tif]

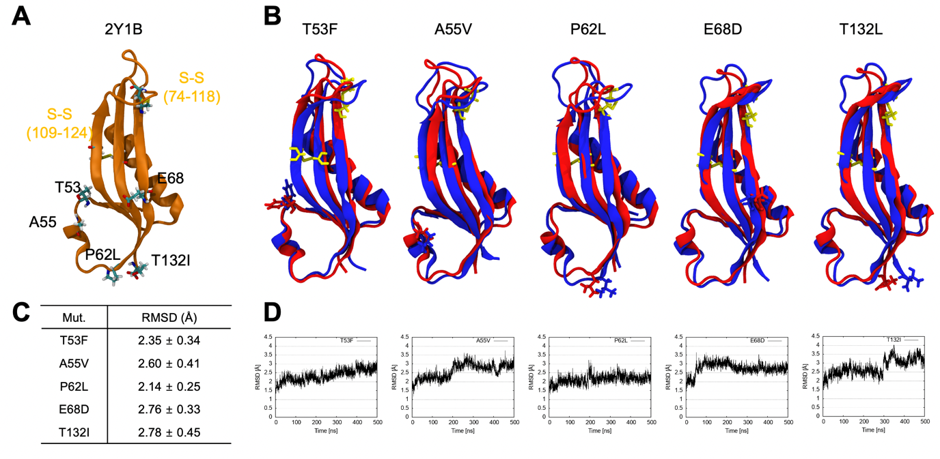

Supplement: S12 Fig — (A) Crystal structure of RcsF (PDB ID 2Y1B). Two non-consecutive disulfide bonds (Cys74−Cys118 and Cys109−Cys124) and the five single-point mutation residues are represented by sticks. (B) Overlaid views of the initial structure (red) and a 500-ns snapshot (blue). There are no distinct changes in the folded structure of the five models. (C) Averaged root-mean-square deviation (RMSD) values of the five models using their 500-ns trajectories show < 3 Å change with respect to the crystal structure. (D) RMSD time series throughout the 500-ns simulation for all five models. (TIF) [file pgen.1010601.s012.tif]

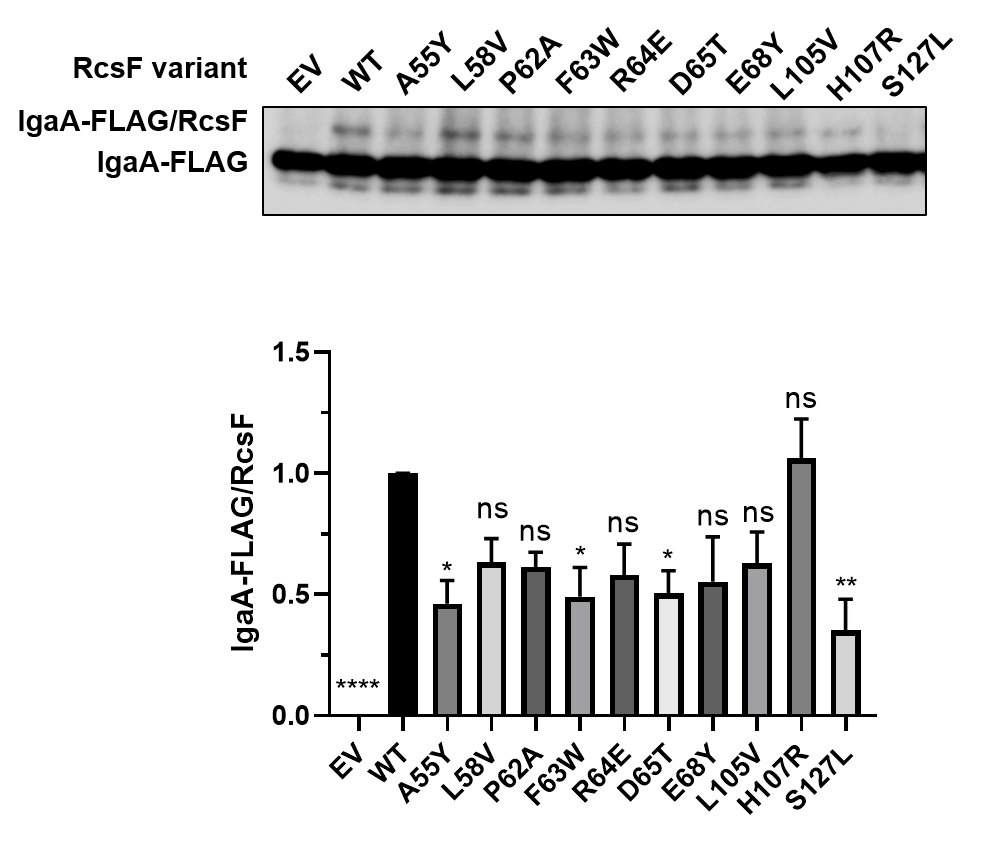

Supplement: S13 Fig — Immunoblot analysis of in vivo formaldehyde crosslinked samples probed with α-FLAG, and quantification of RcsF/IgaA-Flag band relative to the WT. Graphs represent mean of independent experiments +/- SD; n.s. = p ≥ 0.05, * = p < 0.01. (TIF) [file pgen.1010601.s013.tif]

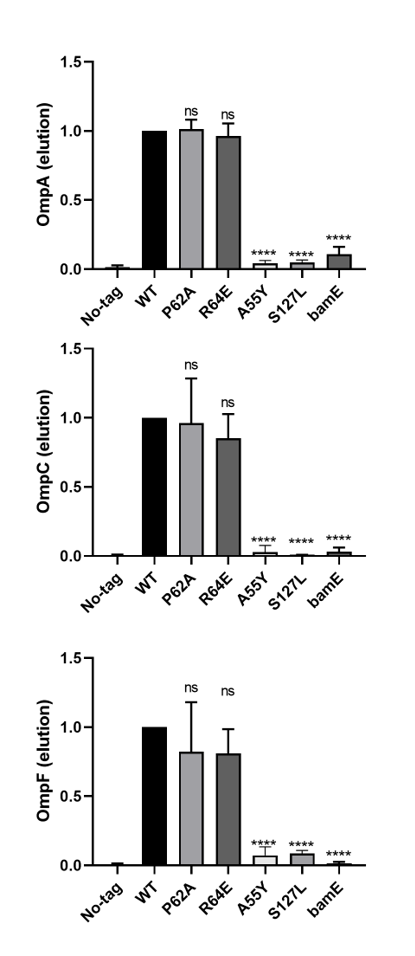

Supplement: S14 Fig — Bands are quantified relative to the WT; mean of independent experiments+/- SD; n.s. = p ≥ 0.05, **** = p < 0.0001. (TIF) [file pgen.1010601.s014.tif]
