# Supplementary material for: Conformational rearrangements in the sensory RcsF/OMP complex mediate signal transduction across the bacterial cell envelope
Source: PLoS Genet. 2023 Jan 27;19(1):e1010601. doi: 10.1371/journal.pgen.1010601 (PMC9907809; doi:10.1371/journal.pgen.1010601)
Supplement: S1 Table — (DOCX) [file pgen.1010601.s015.docx]

**Table S1. Hits from LOF(IM) genetic screen.**

| Targeted residue | Screen hits | Analyzed | Comments |
| --- | --- | --- | --- |
| A55 | K, R, Q, N, D | K | Main text |
|  |  | Q | Same as A55K |
| L58 | R, Q, K, G, H, P, Y, E, S, T, N | Y | Main text |
| P62 | D | D | Main text |
| F63 | R, D, T, P, Q, V, A, E, N, K, Y, G, H, S | Y | Main text |
| R64 | P | - |  |
| D65 | K | K | Main text |
| L66 | P | - |  |
| G71 | K, P, R, C, L | - | G71 not solvent-accessible residue; substitutions disrupt the structure based on in silico mutagenesis. G71 suppressor pool was prescreened by western blot, and RcsF protein not detectable. All variants were complete or partial suppressors in unrelated RcsF(LOF) screen for BamA interaction |
| P82 | R | R | No phenotype in monocultures (upon LolB-depletion) |
| R89 | P | - |  |
| K90 | P | - |  |
| R91 | P | - |  |
| L105 | P, T, D, Q, G, R, E, K, N, A, S, C | A | No RcsF protein |
|  |  | D | No RcsF protein |
|  |  | T | No RcsF protein |
| V123 | P | - |  |
| S127 | K | K | Main text |
| N130 | P | - |  |
