# Supplementary material for: Conformational rearrangements in the sensory RcsF/OMP complex mediate signal transduction across the bacterial cell envelope
Source: PLoS Genet. 2023 Jan 27;19(1):e1010601. doi: 10.1371/journal.pgen.1010601 (PMC9907809; doi:10.1371/journal.pgen.1010601)
Supplement: S2 Table — (DOCX) [file pgen.1010601.s016.docx]

**Table S2. Statistical analysis for β-galactosidase assay data presented in Fig. 3A and B.**

| **Two-way ANOVA** | **Ordinary** |  |  |  |  |  |
| --- | --- | --- | --- | --- | --- | --- |
| **Alpha** | **0.05** |  |  |  |  |  |
|  | Compare each cell mean with the other cell mean in that row | | |  |  |  |
|  |  |  |  |  |  |  |
|  | Number of families | 1 |  |  |  |  |
|  | Number of comparisons per family | 8 |  |  |  |  |
|  | Alpha | 0.05 |  |  |  |  |
|  |  |  |  |  |  |  |
|  | Šídák's multiple comparisons test | Mean Diff. | 95.00% CI of diff. | Below threshold? | Summary | Adjusted P Value |
|  |  |  |  |  |  |  |
|  | **untreated versus PMB** |  |  |  |  |  |
|  | EV | -4.671 | -33.34 to 24.00 | No | ns | 0.9997 |
|  | WT | -90.79 | -119.5 to -62.12 | Yes | **** | <0.0001 |
|  | A55K | -6.64 | -35.31 to 22.03 | No | ns | 0.9968 |
|  | L58Y | -6.223 | -34.89 to 22.44 | No | ns | 0.998 |
|  | P62D | -9.949 | -38.62 to 18.72 | No | ns | 0.9581 |
|  | F63Y | -12.13 | -40.80 to 16.54 | No | ns | 0.8808 |
|  | D65K | -16.9 | -45.56 to 11.77 | No | ns | 0.5668 |
|  | S127K | -5.279 | -33.95 to 23.39 | No | ns | 0.9994 |
|  |  |  |  |  |  |  |
| **Two-way ANOVA** | **Ordinary** |  |  |  |  |  |
| **Alpha** | **0.05** |  |  |  |  |  |
|  | Within each column, compare rows (simple effects within columns) | | |  |  |  |
|  |  |  |  |  |  |  |
|  | Number of families | 3 |  |  |  |  |
|  | Number of comparisons per family | 7 |  |  |  |  |
|  | Alpha | 0.05 |  |  |  |  |
|  |  |  |  |  |  |  |
|  | Dunnett's multiple comparisons test | Mean Diff. | 95.00% CI of diff. | Below threshold? | Summary | Adjusted P Value |
|  |  |  |  |  |  |  |
|  | **IM** |  |  |  |  |  |
|  | WT vs. EV | 198.4 | 161.9 to 234.9 | Yes | **** | <0.0001 |
|  | WT vs. A55K | 191.1 | 154.6 to 227.7 | Yes | **** | <0.0001 |
|  | WT vs. L58Y | 181 | 144.5 to 217.5 | Yes | **** | <0.0001 |
|  | WT vs. P62D | 154 | 117.5 to 190.5 | Yes | **** | <0.0001 |
|  | WT vs. F63Y | 152.2 | 115.7 to 188.8 | Yes | **** | <0.0001 |
|  | WT vs. D65K | 150.5 | 114.0 to 187.0 | Yes | **** | <0.0001 |
|  | WT vs. S127K | 183.5 | 147.0 to 220.0 | Yes | **** | <0.0001 |
|  |  |  |  |  |  |  |
|  | **untreated** |  |  |  |  |  |
|  | WT vs. EV | 104 | 67.51 to 140.5 | Yes | **** | <0.0001 |
|  | WT vs. A55K | 98.69 | 62.18 to 135.2 | Yes | **** | <0.0001 |
|  | WT vs. L58Y | 103 | 66.48 to 139.5 | Yes | **** | <0.0001 |
|  | WT vs. P62D | 103.4 | 66.86 to 139.9 | Yes | **** | <0.0001 |
|  | WT vs. F63Y | 103.9 | 67.38 to 140.4 | Yes | **** | <0.0001 |
|  | WT vs. D65K | 104.1 | 67.56 to 140.6 | Yes | **** | <0.0001 |
|  | WT vs. S127K | 102.8 | 66.28 to 139.3 | Yes | **** | <0.0001 |
|  |  |  |  |  |  |  |
|  | **PMB** |  |  |  |  |  |
|  | WT vs. EV | 190.1 | 153.6 to 226.6 | Yes | **** | <0.0001 |
|  | WT vs. A55K | 182.8 | 146.3 to 219.3 | Yes | **** | <0.0001 |
|  | WT vs. L58Y | 187.5 | 151.0 to 224.1 | Yes | **** | <0.0001 |
|  | WT vs. P62D | 184.2 | 147.7 to 220.7 | Yes | **** | <0.0001 |
|  | WT vs. F63Y | 182.5 | 146.0 to 219.1 | Yes | **** | <0.0001 |
|  | WT vs. D65K | 178 | 141.4 to 214.5 | Yes | **** | <0.0001 |
|  | WT vs. S127K | 188.3 | 151.8 to 224.8 | Yes | **** | <0.0001 |
