# Supplementary material for: Conformational rearrangements in the sensory RcsF/OMP complex mediate signal transduction across the bacterial cell envelope
Source: PLoS Genet. 2023 Jan 27;19(1):e1010601. doi: 10.1371/journal.pgen.1010601 (PMC9907809; doi:10.1371/journal.pgen.1010601)
Supplement: S3 Table — (DOCX) [file pgen.1010601.s017.docx]

**Table S3. Hits from GOF(OM) the genetic screen.**

| Targeted residue | Screen hits | Analyzed | Comments |
| --- | --- | --- | --- |
| T53 | F,W | F | Main text |
| N54 | E | E | Did not increase P*rprA-lacZ* activity |
| A55 | V, I | V | Main text |
| P62 | L, M | L | Main text |
| D65 | F | F | P*rprA-lacZ* activity increased but <2 fold |
| L66 | R | R | P*rprA-lacZ* increased but <2 fold |
| E68 | D | D | Main text |
| S77 | L | L | P*rprA-lacZ* increased but <2 fold |
| N78 | C | C | P*rprA-lacZ* g increased 3 fold, but forms intramolecular crosslinks |
| L105 | W | W | P*rprA-lacZ* increased but <2 fold |
| T132 | I, L, M, V | I | Main text |
