# Supplementary material for: Conformational rearrangements in the sensory RcsF/OMP complex mediate signal transduction across the bacterial cell envelope
Source: PLoS Genet. 2023 Jan 27;19(1):e1010601. doi: 10.1371/journal.pgen.1010601 (PMC9907809; doi:10.1371/journal.pgen.1010601)
Supplement: S4 Table — (DOCX) [file pgen.1010601.s018.docx]

**Table S4.** **Statistical analysis for β-galactosidase assay data presented in Fig. 4E**

| **Strain** | **untreated** | | **PMB** |  | **Fold change** | **2-way ANOVA,** | |  |  |  |
| --- | --- | --- | --- | --- | --- | --- | --- | --- | --- | --- |
|  | Mean | SD | Mean | SD | (PMB/untreated) | Mean Diff. | 95.00% CI of diff. | Below threshold? | Summary | Adjusted P Value |
| EV | 15.30 | 8.39 | 15.20 | 2.85 | 0.99 | -18.86 | -114.5 to 76.81 | No | ns | >0.9999 |
| WT | 101.39 | 30.69 | 242.85 | 70.72 | 2.40 | -116.3 | -212.0 to -20.61 | Yes | ** | 0.0062 |
| T53F | 178.09 | 15.92 | 267.06 | 43.00 | 1.50 | -88.98 | -184.7 to 6.693 | No | ns | 0.0912 |
| A55V | 229.30 | 24.16 | 330.65 | 40.52 | 1.44 | -101.3 | -197.0 to -5.677 | Yes | * | 0.029 |
| P62L | 278.55 | 40.40 | 364.30 | 44.50 | 1.31 | -85.75 | -181.4 to 9.922 | No | ns | 0.1198 |
| E68D | 236.35 | 33.23 | 347.98 | 37.84 | 1.47 | -111.6 | -207.3 to -15.96 | Yes | * | 0.0101 |
| T132I | 326.29 | 47.44 | 413.34 | 25.91 | 1.27 | -87.04 | -182.7 to 8.628 | No | ns | 0.1076 |
