# Supplementary material for: Conformational rearrangements in the sensory RcsF/OMP complex mediate signal transduction across the bacterial cell envelope
Source: PLoS Genet. 2023 Jan 27;19(1):e1010601. doi: 10.1371/journal.pgen.1010601 (PMC9907809; doi:10.1371/journal.pgen.1010601)
Supplement: S5 Table — These hits are non-Proline mutants and behaved like WT in the LOF(IM) screen. (DOCX) [file pgen.1010601.s019.docx]

**Table S5. Hits from LOF(OM) genetic screen. These hits are non-Proline mutants and behaved like WT in the LOF(IM) screen.**

| Targeted residue | Screen hits | Analyzed | Comments |
| --- | --- | --- | --- |
| I51 | R, K, D, G | R | Reduced RcsF protein |
| Y52 | R, K, D, E, Q | R | Reduced RcsF protein |
| A55 | Y, G, S, W | Y | Main text |
|  |  | G | Similar to A55Y at the OM, reduced RcsF levels at the IM |
| L58 | A, V, C | V | Main text |
| V59 | K | K | Reduced RcsF protein |
| G60 | T, K, I, V, W, I, C | T | Reduced RcsF protein |
| P62 | A, Y, E, S, N, H, G | A | Main text |
| F63 | W | W | Main text |
| R64 | E, D, C, T, S, A, G | E | Main text |
| D65 | T, G, Q | T | Main text |
| L66 | D | D | Reduced RcsF protein |
| G67 | W, F, L, Y, E | W | Reduced RcsF protein |
| E68 | Y, W, C | Y | Main text |
| G71 | I, Q, V, T, Y | - | G71 not solvent-accessible residue; substitutions disrupt the struc­ture based on in silico mutagenesis. |
| N102 | W, L, I, C | W | Only partially active at the IM |
| L105 | V | V | Main text |
| H107 | K,R, C | R | Main text |
| V123 | C | - | Not pursued because extra Cys causes abnormal disulfides |
| S127 | L, H, D, C | L | Main text |
