# Supplementary material for: Conformational rearrangements in the sensory RcsF/OMP complex mediate signal transduction across the bacterial cell envelope
Source: PLoS Genet. 2023 Jan 27;19(1):e1010601. doi: 10.1371/journal.pgen.1010601 (PMC9907809; doi:10.1371/journal.pgen.1010601)
Supplement: S6 Table — (DOCX) [file pgen.1010601.s020.docx]

**Table S6. Statistical analysis for β-galactosidase assay data presented in Fig. 5 A and B.**

| **Two-way ANOVA** | **Ordinary** |  |  |  |  |
| --- | --- | --- | --- | --- | --- |
| **Alpha** | **0.05** |  |  |  |  |
|  |  |  |  |  |  |
| Within each column, compare rows (simple effects within columns) | | |  |  |  |
|  |  |  |  |  |  |
| Number of families | 3 |  |  |  |  |
| Number of comparisons per family | 11 |  |  |  |  |
| Alpha | 0.05 |  |  |  |  |
|  |  |  |  |  |  |
| Dunnett's multiple comparisons test | Mean Diff. | 95.00% CI of diff. | Below threshold? | Summary | Adjusted P Value |
|  |  |  |  |  |  |
| **untreated** |  |  |  |  |  |
| WT vs. EV | 55.13 | 22.66 to 87.60 | Yes | **** | <0.0001 |
| WT vs. A55Y | 56 | 23.53 to 88.47 | Yes | **** | <0.0001 |
| WT vs. L58V | 50.77 | 18.30 to 83.24 | Yes | *** | 0.0004 |
| WT vs. P62A | 51.47 | 19.00 to 83.94 | Yes | *** | 0.0003 |
| WT vs. F63W | 54.63 | 22.16 to 87.10 | Yes | *** | 0.0001 |
| WT vs. R64E | 52.97 | 20.50 to 85.44 | Yes | *** | 0.0002 |
| WT vs. D65T | 52.97 | 20.50 to 85.44 | Yes | *** | 0.0002 |
| WT vs. E68Y | 52.57 | 20.10 to 85.04 | Yes | *** | 0.0002 |
| WT vs. L105V | 53.2 | 20.73 to 85.67 | Yes | *** | 0.0002 |
| WT vs. H107R | 54 | 21.53 to 86.47 | Yes | *** | 0.0001 |
| WT vs. S127L | 52.13 | 19.66 to 84.60 | Yes | *** | 0.0003 |
|  |  |  |  |  |  |
| **PMB** |  |  |  |  |  |
| WT vs. EV | 99.9 | 67.43 to 132.4 | Yes | **** | <0.0001 |
| WT vs. A55Y | 89.53 | 57.06 to 122.0 | Yes | **** | <0.0001 |
| WT vs. L58V | 75.37 | 42.90 to 107.8 | Yes | **** | <0.0001 |
| WT vs. P62A | 76.97 | 44.50 to 109.4 | Yes | **** | <0.0001 |
| WT vs. F63W | 86.67 | 54.20 to 119.1 | Yes | **** | <0.0001 |
| WT vs. R64E | 82.1 | 49.63 to 114.6 | Yes | **** | <0.0001 |
| WT vs. D65T | 81.3 | 48.83 to 113.8 | Yes | **** | <0.0001 |
| WT vs. E68Y | 75.7 | 43.23 to 108.2 | Yes | **** | <0.0001 |
| WT vs. L105V | 77.27 | 44.80 to 109.7 | Yes | **** | <0.0001 |
| WT vs. H107R | 69.67 | 37.20 to 102.1 | Yes | **** | <0.0001 |
| WT vs. S127L | 57.53 | 25.06 to 90.00 | Yes | **** | <0.0001 |
|  |  |  |  |  |  |
| **Two-way ANOVA** | **Ordinary** |  |  |  |  |
| **Alpha** | **0.05** |  |  |  |  |
|  |  |  |  |  |  |
| Compare each cell mean with the other cell mean in that row | | |  |  |  |
|  |  |  |  |  |  |
| Number of families | 1 |  |  |  |  |
| Number of comparisons per family | 12 |  |  |  |  |
| Alpha | 0.05 |  |  |  |  |
|  |  |  |  |  |  |
| Šídák's multiple comparisons test | Mean Diff. | 95.00% CI of diff. | Below threshold? | Summary | Adjusted P Value |
|  |  |  |  |  |  |
| **untreated versus PMB** |  |  |  |  |  |
| EV | -1.87 | -23.29 to 19.55 | No | ns | >0.9999 |
| WT | -46.63 | -68.05 to -25.22 | Yes | **** | <0.0001 |
| A55Y | -13.1 | -34.52 to 8.316 | No | ns | 0.5955 |
| L58V | -22.03 | -43.45 to -0.6175 | Yes | * | 0.0395 |
| P62A | -21.13 | -42.55 to 0.2825 | No | ns | 0.0556 |
| F63W | -14.6 | -36.02 to 6.816 | No | ns | 0.4338 |
| R64E | -17.5 | -38.92 to 3.916 | No | ns | 0.1949 |
| D65T | -18.3 | -39.72 to 3.116 | No | ns | 0.1508 |
| E68Y | -23.5 | -44.92 to -2.084 | Yes | * | 0.0222 |
| L105V | -22.57 | -43.98 to -1.151 | Yes | * | 0.0321 |
| H107R | -30.97 | -52.38 to -9.551 | Yes | *** | 0.0009 |
| S127L | -41.23 | -62.65 to -19.82 | Yes | **** | <0.0001 |
