# Supplementary material for: Conformational rearrangements in the sensory RcsF/OMP complex mediate signal transduction across the bacterial cell envelope
Source: PLoS Genet. 2023 Jan 27;19(1):e1010601. doi: 10.1371/journal.pgen.1010601 (PMC9907809; doi:10.1371/journal.pgen.1010601)
Supplement: S7 Table — Unless otherwise indicated, the host background is MC4100 (JCM158). (DOCX) [file pgen.1010601.s021.docx]

**Table S7. Strains used in this study.** Unless otherwise indicated, the host background is MC4100 (JCM158)

| **Strain** | **Genotype** | **Reference** |
| --- | --- | --- |
| Mach-1 | F- *φ80(lacZ)∆M15 ∆lacX74 hsdR(rK-mK+) ∆recA1398 endA1 tonA* | Invitrogen |
| pGP-Tn7-Gn | S17-1(λ attpir) pGP-Tn7-Gn | [1] |
| pSTNSK | pST76-K::tnsABCD | [1] |
| pBAD18::*rcsF*_SM/DQ | S17D M18Q, Lol-avoidance mutant | [2] |
| pZS21 |  | [3] |
| DH300 | MG1655 *Δlac PrprA-lacZYA* | [4] |
| JCM158 | MC4100 *araR*/- | [5] |
| MG2201 | *ΔlolB, Δlpp, ΔrcsF, pBAD18::lolB* | [6] |
| AK-265 | *λ att (PrprA-lacZYA)* | [7] |
| AK-266 | *ΔrcsF λ att (PrprA-lacZYA)* | [7] |
| AK-688 | *ΔrcsF ΔbamE λ att (PrprA-lacZYA)* | [8] |
| SL-71 | MG2201 pZS21 | This study |
| SL-72 | MG2201 pZS21::rscF | This study |
| SL-81 | MG2201 pZS21:: *rcsF* L58Y | This study |
| SL-82 | MG2201 pZS21:: *rcsF* F63Y | This study |
| SL-106 | MG2201 pZS21::*rcsF* A55K | This study |
| SL-108 | MG2201 pZS21::*rcsF* P62D | This study |
| SL-140 | MG2201 pZS21::*rcsF* D65K | This study |
| SL-142 | MG2201 pZS21::*rcsF* S127K | This study |
| AK-663 | AK-266 pZS21::*rcsF* | [7] |
| AK-680 | AK-266 pZS21 | [7] |
| SL-101 | AK-266 pZS21::*rcsF* A55K | This study |
| AK-682 | AK-266 pZS21:: *rcsF* L58Y | This study |
| SL-103 | AK-266 pZS21::*rcsF* P62D | This study |
| AK-683 | AK-266 pZS21:: *rcsF* F63Y | This study |
| SL-134 | AK-266 pZS21::*rcsF* D65K | This study |
| SL-136 | AK-266 pZS21::*rcsF* S127K | This study |
| SL-169 | AK-266 pZS21::*rcsF* T53F | This study |
| SL-171 | AK-266 pZS21::*rcsF* A55V | This study |
| SL-172 | AK-266 pZS21::*rcsF* P62L | This study |
| SL-175 | AK-266 pZS21::*rcsF* E68D | This study |
| SL-178 | AK-266 pZS21::*rcsF* T132i | This study |
| AK-681 | AK-266 pZS21:: *rcsF* A55Y | [7] |
| AK-1494 | AK-266 pZS21::*rcsF* L58V | This study |
| AK-1498 | AK-266 pZS21::*rcsF* P62A | This study |
| AK-1499 | AK-266 pZS21::*rcsF* F63W | This study |
| AK-1500 | AK-266 pZS21::*rcsF* R64E | This study |
| AK-1501 | AK-266 pZS21::*rcsF* D65T | This study |
| AK-1505 | AK-266 pZS21::*rcsF* E68Y | This study |
| AK-1577 | AK-266 pZS21::*rcsF* L105V | This study |
| AK-1578 | AK-266 pZS21::*rcsF* H107R | This study |
| AK-1506 | AK-266 pZS21::*rcsF* S127L | This study |
| AK-1540 | AK-266 pBAD18 | This study |
| AK-1541 | AK-266 PBAD18::*rcsF* WT | This study |
| AK-1542 | AK-266 PBAD18::*rcsF* SM/DQ | This study |
| AK-1544 | AK-266 PBAD18::*rcsF* SM/DQ A55Y | This study |
| AK-1545 | AK-266 PBAD18::*rcsF* SM/DQ L58V | This study |
| AK-1548 | AK-266 PBAD18::*rcsF* SM/DQ P62A | This study |
| AK-1549 | AK-266 PBAD18::*rcsF* SM/DQ F63W | This study |
| AK-1550 | AK-266 PBAD18::*rcsF* SM/DQ R64E | This study |
| AK-1551 | AK-266 PBAD18::*rcsF* SM/DQ D65T | This study |
| AK-1552 | AK-266 PBAD18::*rcsF* SM/DQ E68Y | This study |
| AK-1554 | AK-266 PBAD18::*rcsF* SM/DQ L105V | This study |
| AK-1555 | AK-266 PBAD18::*rcsF* SM/DQ H107R | This study |
| AK-1556 | AK-266 PBAD18::*rcsF* SM/DQ S127L | This study |
| SL-242 | AK-266 pBAD18::*rcsF*_SM/DQ A55K | This study |
| SL-244 | AK-266 pBAD18::*rcsF*_SM/DQ L58Y | This study |
| SL-245 | AK-266 pBAD18::*rcsF*_SM/DQ P62D | This study |
| SL-246 | AK-266 pBAD18::*rcsF*_SM/DQ F63Y | This study |
| SL-247 | AK-266 pBAD18::*rcsF*_SM/DQ D65K | This study |
| SL-248 | AK-266 pBAD18::*rcsF*_SM/DQ S127K | This study |
| SL-249 | AK-266 pBAD18::*rcsF*_SM/DQ T53F | This study |
| SL-250 | AK-266 pBAD18::*rcsF*_SM/DQ A55V | This study |
| SL-251 | AK-266 pBAD18::*rcsF*_SM/DQ P62L | This study |
| SL-252 | AK-266 pBAD18::*rcsF*_SM/DQ E68D | This study |
| SL-253 | AK-266 pBAD18::*rcsF*_SM/DQ T132i | This study |
| SL-199 | AK-266 p-*igaA*-FLAG | This study |
| SL-208 | AK-266 p-*igaA*-FLAG pZS21 | This study |
| SL-209 | AK-266 p-*igaA*-FLAG pZS21::*rcsF* | This study |
| SL-211 | AK-266 p-*igaA*-FLAG pZS21::*rcsF* A55K | This study |
| SL-213 | AK-266 p-*igaA*-FLAG pZS21::*rcsF* P62D | This study |
| SL-214 | AK-266 p-*igaA*-FLAG pZS21::*rcsF* D65K | This study |
| SL-215 | AK-266 p-*igaA*-FLAG pZS21::*rcsF* S127K | This study |
| SL-218 | AK-266 p-*igaA*-FLAG pZS21::*rcsF* L58Y | This study |
| SL-219 | AK-266 p-*igaA*-FLAG pZS21::*rcsF* F63Y | This study |
| SL-220 | AK-266 p-*igaA*-FLAG pZS21::*rcsF* T53F | This study |
| SL-221 | AK-266 p-*igaA*-FLAG pZS21::*rcsF* A55V | This study |
| SL-222 | AK-266 p-*igaA*-FLAG pZS21::*rcsF* P62L | This study |
| SL-225 | AK-266 p-*igaA*-FLAG pZS21::*rcsF* T132i | This study |
| SL-226 | AK-266 p-*igaA*-FLAG pZS21::*rcsF* E68D | This study |
| AK-666 | AK-266 pZS21::*rcsF*-Strep | This study |
| AK-895 | AK-688 pZS21::*rcsF*-strep | This study |
| SK-16 | AK-266 pZS21::*rcsF*(A55Y)-Strep | This study |
| SK-109 | AK-266 pZS21::*rcsF* (S127L)-Strep | This study |
| SK-119 | AK-266 pZS21::*rcsF*(T53F)-Strep | This study |
| SK-120 | AK-266 pZS21::*rcsF*(P62A)-Strep | This study |
| SK-121 | AK-266 pZS21::*rcsF*(R64E)-Strep | This study |
| SK-122 | AK-266 pZS21::*rcsF*(A55V)-Strep | This study |
| SL-62 | AK-266 *ΔigaA malT::Tn10* attTn7::*igaA-FLAG* | This study |
| SL-66 | SL-62 pBAD18 | This study |
| SL-189 | SL-62 pBAD18::*rcsF* SM/DQ | This study |
| SL-190 | SL-62 pBAD18::*rcsF* SM/DQ A55K | This study |
| SL-192 | SL-62 pBAD18::*rcsF* SM/DQ L58Y | This study |
| SL-193 | SL-62 pBAD18::*rcsF* SM/DQ P62D | This study |
| SL-195 | SL-62 pBAD18::*rcsF* SM/DQ F63Y | This study |
| SL-196 | SL-62 pBAD18::*rcsF* SM/DQ D65K | This study |
| SL-197 | SL-62 pBAD18::*rcsF* SM/DQ S127K | This study |
| SL-257 | SL-62 pBAD18::*rcsF* SM/DQ T53F | This study |
| SL-258 | SL-62 pBAD18::*rcsF* SM/DQ A55V | This study |
| SL-259 | SL-62 pBAD18::*rcsF* SM/DQ P62L | This study |
| SL-261 | SL-62 pBAD18::*rcsF* SM/DQ E68D | This study |
| SL-262 | SL-62 pBAD18::*rcsF* SM/DQ T132I | This study |
| AK-1584 | SL-62 pBAD18::*rcsF* SM/DQ A55Y | This study |
| AK-1585 | SL-62 pBAD18::*rcsF* SM/DQ L58V | This study |
| AK-1586 | SL-62 pBAD18::*rcsF* SM/DQ P62A | This study |
| AK-1587 | SL-62 pBAD18::*rcsF* SM/DQ F63W | This study |
| AK-1588 | SL-62 pBAD18::*rcsF* SM/DQ R64E | This study |
| AK-1589 | SL-62 pBAD18::*rcsF* SM/DQ D65T | This study |
| AK-1590 | SL-62 pBAD18::*rcsF* SM/DQ E68Y | This study |
| AK-1591 | SL-62 pBAD18::*rcsF* SM/DQ L105V | This study |
| AK-1592 | SL-62 pBAD18::*rcsF* SM/DQ H107R | This study |
| AK-1593 | SL-62 pBAD18::*rcsF* SM/DQ S127L | This study |
| AK-1596 | AK-266 p-*igaA*-FLAG pZS21::*rcsF* A55Y | This study |
| AK-1597 | AK-266 p-*igaA*-FLAG pZS21::*rcsF* L58V | This study |
| AK-1598 | AK-266 p-*igaA*-FLAG pZS21::*rcsF* P62A | This study |
| AK-1599 | AK-266 p-*igaA*-FLAG pZS21::*rcsF* F63W | This study |
| AK-1600 | AK-266 p-*igaA*-FLAG pZS21::*rcsF* R64E | This study |
| AK-1601 | AK-266 p-*igaA*-FLAG pZS21::*rcsF* D65T | This study |
| AK-1602 | AK-266 p-*igaA*-FLAG pZS21::*rcsF* E68Y | This study |
| AK-1603 | AK-266 p-*igaA*-FLAG pZS21::*rcsF* L105V | This study |
| AK-1604 | AK-266 p-*igaA*-FLAG pZS21::*rcsF* H107R | This study |
| AK-1605 | AK-266 p-*igaA*-FLAG pZS21::*rcsF* S127L | This study |

**References**

1. Crepin S, Harel J, Dozois CM. Chromosomal complementation using Tn7 transposon vectors in Enterobacteriaceae. Appl Environ Microbiol. 2012;78(17):6001-8. Epub 2012/06/19. doi: 10.1128/AEM.00986-12. PubMed PMID: 22706059; PubMed Central PMCID: PMCPMC3416591.

2. Konovalova A, Perlman DH, Cowles CE, Silhavy TJ. Transmembrane domain of surface-exposed outer membrane lipoprotein RcsF is threaded through the lumen of β-barrel proteins. Proceedings of the National Academy of Sciences of the United States of America. 2014;111(41):E4350-E8. doi: 10.1073/pnas.1417138111. PubMed PMID: WOS:000342922000013.

3. Lutz R, Bujard H. Independent and tight regulation of transcriptional units in *Escherichia coli* via LacR/O, the TetR/O and AraC/I1-2 regulatory elements. Nucl Acids Res. 1997;25:1203-10.

4. Majdalani N, Chen S, Murrow J, St John K, Gottesman S. Regulation of RpoS by a novel small RNA: the characterization of RprA. Mol Microbiol. 2001;39(5):1382-94. Epub 2001/03/17. doi: 10.1111/j.1365-2958.2001.02329.x. PubMed PMID: 11251852.

5. Malinverni JC, Werner J, Kim S, Sklar JG, Kahne D, Misra R, et al. YfiO stabilizes the YaeT complex and is essential for outer membrane protein assembly in Escherichia coli. Mol Microbiol. 2006;61(1):151-64. Epub 2006/07/11. doi: 10.1111/j.1365-2958.2006.05211.x. PubMed PMID: 16824102.

6. Grabowicz M, Silhavy TJ. Redefining the essential trafficking pathway for outer membrane lipoproteins. Proc Natl Acad Sci U S A. 2017;114(18):4769-74. Epub 2017/04/19. doi: 10.1073/pnas.1702248114. PubMed PMID: 28416660; PubMed Central PMCID: PMCPMC5422772.

7. Konovalova A, Mitchell AM, Silhavy TJ. A lipoprotein/beta-barrel complex monitors lipopolysaccharide integrity transducing information across the outer membrane. Elife. 2016;5. Epub 2016/06/11. doi: 10.7554/eLife.15276. PubMed PMID: 27282389; PubMed Central PMCID: PMCPMC4942254.

8. Tata M, Konovalova A. Improper Coordination of BamA and BamD Results in Bam Complex Jamming by a Lipoprotein Substrate. mBio. 2019;10(3). Epub 2019/05/23. doi: 10.1128/mBio.00660-19. PubMed PMID: 31113900; PubMed Central PMCID: PMCPMC6529637.
