# Supplementary material for: Conformational rearrangements in the sensory RcsF/OMP complex mediate signal transduction across the bacterial cell envelope
Source: PLoS Genet. 2023 Jan 27;19(1):e1010601. doi: 10.1371/journal.pgen.1010601 (PMC9907809; doi:10.1371/journal.pgen.1010601)
Supplement: S8 Table — (DOCX) [file pgen.1010601.s022.docx]

**Table S8. Primers used in this study**

| **Construct** | **primer#** | **Primer sequence** |
| --- | --- | --- |
| A55K | SL-75 | AAA GAA GAA TTA GTC GGC AAA CCG TTC CGC |
|  | AK-789 | ATTGGTATAAATTCGGACCGGCGTGGC |
| L58Y | SL-92 | TAC GTC GGC AAA CCG TTC CGC |
|  | AK-792 | TTC TTC TGC ATT GGT ATA AAT TCG GAC CGG |
| P62D | SL-78 | GAT TTC CGC GAT CTC GGT GAA GTC AG |
|  | AK-796 | TTT GCC GAC TAA TTC TTC TGC ATT GGT ATA AAT TCG |
| F63Y | SL-91 | TAC CGC GAT CTC GGT GAA GTC AGT G |
|  | AK-797 | CGG TTT GCC GAC TAA TTC TTC TGC ATT GG |
| D65K | SL-81 | AAA CTC GGT GAA GTC AGT GGC GAC |
|  | AK-799 | GCG GAA CGG TTT GCC GAC TAA TTC TTC |
| S127K | SL-86 | AAA GCG CTT AAC ATT ACG GCG AAA TGA |
|  | AK-551 | ACC GAT ACA TAC AGC CTG ACG ATA GC |
| T53F | AK-1013 | TTC AAT GCA GAA GAA TTA GTC GGC AAA CCG |
|  | AK-787 | ATA AAT TCG GAC CGG CGT GGC |
| A55V | AK-1015 | GTG GAA GAA TTA GTC GGC AAA CCG TTC CGC |
|  | AK-789 | ATTGGTATAAATTCGGACCGGCGTGGC |
| P62L | AK-1016 | CTG TTC CGC GAT CTC GGT GAA GTC AG |
|  | AK-796 | TTT GCC GAC TAA TTC TTC TGC ATT GGT ATA AAT TCG |
| E68D | AK-1025 | GAT GTC AGT GGC GAC TCT TGC CAG |
|  | AK-802 | ACC GAG ATC GCG GAA CGG TTT G |
| T132I (pZS21) | AK-1024 | ATT GCG AAA TGA GCG GTA CCC G |
|  | AK-833 | AAT GTT AAG CGC AGA ACC GAT ACA TAC AGC |
| T132I (pBAD18) | SL-93 | ATTGCGAAATGAtctagagtcgacctg |
|  | AK-833 | AAT GTT AAG CGC AGA ACC GAT ACA TAC AGC |
| L58V | AK-1035 | GTG GTC GGC AAA CCG TTC CGC |
|  | AK-792 | TTC TTC TGC ATT GGT ATA AAT TCG GAC CGG |
| P62A | AK-1039 | GCG TTC CGC GAT CTC GGT GAA GTC AG |
|  | AK-796 | TTT GCC GAC TAA TTC TTC TGC ATT GGT ATA AAT TCG |
| F63W | AK-1040 | TGG CGC GAT CTC GGT GAA GTC AGT G |
|  | AK-797 | CGG TTT GCC GAC TAA TTC TTC TGC ATT GG |
| R64E | AK-1041 | GAA GAT CTC GGT GAA GTC AGT GGC GAC |
|  | AK-798 | GAA CGG TTT GCC GAC TAA TTC TTC TGC |
| D65T | AK-1042 | ACG CTC GGT GAA GTC AGT GGC GAC |
|  | AK-799 | GCG GAA CGG TTT GCC GAC TAA TTC TTC |
| E68Y | AK-1046 | TAT GTC AGT GGC GAC TCT TGC CAG |
|  | AK-802 | ACC GAG ATC GCG GAA CGG TTT G |
| L105V | AK-1048 | GTG CTG CAT AGC TGC GAA GTC ACC |
|  | AK-529 | TAC AGC ATT GGC TTT CAT TTT AGA GGC GTT G |
| H107R | AK-1049 | CGT AGC TGC GAA GTC ACC AGC |
|  | AK-530 | CAGTAATACAGCATTGGCTTTCATTTTAGAGGC |
| S127L | AK-1050 | CTG GCG CTT AAC ATT ACG GCG AAA TGA |
|  | AK-551 | ACC GAT ACA TAC AGC CTG ACG ATA GC |
| p-igaA | AK-677 | ATCGC gagctc CGA TAT CGG ACA CGC TTT CGG CAA TGT G |
|  | AK-679 | ATCGCcccgggTTCGATAAGGCTTTCTGAAGGGGTGATCAGTTGC |
| linker-3xFLAG gene block | AK-680 | CGATcccgggGGTGGAGGTTCCGGAGGTGGATCGGGAGGTTCG GACTACAAAGACCATGACGGTGATTATAAAGATCATGATATCGATTACAAGGATGACGATGACAAGTGATGActcgagAAGCTTATCG |
| igaA::Kan recombineering | SL-1 | AGGGTAGCATAACCTGCCGCGCAAACGTGTTATTCGATAAGGCTTTCTGAATTCCGGGGATCCGTCGACC |
|  | SL-2 | CCGGTGCGACTGACCACGCCTGACAGACTAAGTAAGATGGGGAAAGCATGTGTAGGCTGGAGCTGCTTCG |
