# Supplementary material for: Conformational rearrangements in the sensory RcsF/OMP complex mediate signal transduction across the bacterial cell envelope
Source: PLoS Genet. 2023 Jan 27;19(1):e1010601. doi: 10.1371/journal.pgen.1010601 (PMC9907809; doi:10.1371/journal.pgen.1010601)
Supplement: S1 Materials and Methods — (DOCX) [file pgen.1010601.s023.docx]

**Supplemental Materials and Methods**

**Construction of *rcsF* mutants.**

The construction of the *rcsF* mutant library was described in detail [1]. All *rcsF* mutant hits were rebuilt *de novo* using site-directed mutagenesis (SDM) using Q5 polymerase (NEB), and pZS21::*rcsF,* pZS21::*rcsF-Strep* or pBAD18*::rcsF_SM/DQ* as templates. Primers used for SDM are listed in Table S8. The purified PCR product (100 ng) was subjected to *in vitro* phosphorylation/ligation using T4 Polynucleotide Kinase, T4 DNA ligase, and DpnI (NEB), and transformed into *E. coli* Mach1 competent cells. Plasmids were sequenced and transformed into a relevant genetic background.

**Construction of chromosomal and plasmid constructs encoding for IgaA-FLAG.**

*igaA,* together with 418 bp upstream region that covers all putative *igaA* promoters (EcoCyc.org), was amplified with AK-677 and 679 from genomic DNA, and cloned together with the gene block encoding for the serine-glycine linker with 3xFLAG (amino acid sequence PGGGGSGGGSGGSDYKDHDGDYKDHDIDYKDDDDK) resulting in the translational C-terminal fusion of *igaA* with 3xFLAG. The resulting fragment was subcloned into pGP-Tn7-Gn plasmid [2] using the NEB HiFi DNA Assembly. The *igaA-FLAG* construct and an empty vector were introduced into the chromosomal *attTn7* site using a published protocol [3], and verified by colony PCR and sequencing. To test for functionality of the construct, P1 co-transduction frequency of Δ*igaA::Kan* and *malT::Tn10* was compared to the *ΔrcsB* strain, in which *igaA* is no longer essential (Fig. S4A), followed by β-galactosidase assay (Fig. S4B). Since we could not detect RcsF/IgaA complex by crosslinking in this background consistent with other studies [4], we subcloned the same fragment into the pBR322 vector backbone derived from pBAD18, generating *p-igaA-FLAG* plasmid which was then used for crosslinking studies.

**RcsF/IgaA complex structure prediction.**

ColabFold was used to generate structural predictions [5]. Amino acid sequences of the RcsF protein devoid its signal sequence and the lipid-modified cysteine residue together with the full length IgaA protein were analyzed using default settings (“protein structure prediction using "AlphaFold2-ptm" and complex prediction "AlphaFold-multimer-v2". For complexes "AlphaFold-multimer-v[1,2]" and "AlphaFold-ptm" can be used”) at the Google Collab interface (<https://colab.research.google.com/github/sokrypton/ColabFold/blob/main/AlphaFold2.ipynb>). All five predicted models yielded similar results; the top scoring structural model was used to generate Fig. S6.

**RcsF/OMP complex modeling.**

To develop RcsF-OMP complex models, two types of complexes, RcsF/OmpC and RcsF/OmpF, were generated as follows: (1) the N-terminus (N*t*) of RcsF (PDB ID 2Y1B) was placed in the periplasmic region of one OmpC (PDB ID 2J1N) or OmpF (PDB ID 2OMF) barrel, and the N*t* was pulled gently along the *Z* axis through the restrained OMP barrel until the disordered proline-rich linker of RcsF (residues 17−48) was outside the barrel. (2) Cys^16^ (N*t*) was lipidated with the CYS-linker, and the position of acyl tails of the CYS-linker were adjusted on the same lateral position of the hydrophobic region of the outer leaflet of the outer membrane. (3) RcsF/OMP complexes were assembled with the *E. coli* K12 outer membrane using CHARMM-GUI Membrane Builder [6]. (4) the 100-ns NPT simulations with two 5 Å distance restraints (100 kJ・mol^-1^・nm^-2^) between G103^OmpC^-G60^RcsF^ and A123^OmpC^-G60^RcsF^ or Y22^OmpF^-G60^RcsF^ and E109^OmpF^-G60^RcsF^ were performed for initial RcsF-OMP complex model generation and to explore potential interactions between G60^RcsF^ and its neighboring OMP residues [7]. (5) additional 100-ns NPT simulations without any restraint were performed.

**MD simulations of RcsF GOF(OM) variants.**

Five single mutation models of RcsF (T53F, A55V, P62L, E68D, T132I) were prepared to examine the effect of point mutation substitution on the structure. Each single mutation model was generated following the mutation protocol for solution systems in CHARMM-GUI [8,9] with the CHARMM36 force field, and they were placed in the TIP3P [10] water box containing 150 mM KCl bulk ions. After a short energy minimization and 125-ps equilibration with positional restraints for protein backbone (400 kJ・mol^-1^・nm^-2^) and side chain (40 kJ・mol^-1^・nm^-2^), 500-ns NPT (isobaric and isothermal) simulations [11] with hydrogen mass repartitioning (HMR) were performed with 4-fs time step at 310.15 K and 1 bar pressure using the OpenMM-7.5.0 package [12]. The particle-mesh Ewald method [13] for long-range electrostatic interactions and the force-switch method (10−12 Å) for van der Waals interactions were applied, and temperature and pressure were maintained using the temperature coupling and isotropic Monte Carlo barostat method.

References

1. Tata M, Kumar S, Lach SR, Saha S, Hart EM, Konovalova A. High-throughput suppressor screen demonstrates that RcsF monitors outer membrane integrity and not Bam complex function. Proc Natl Acad Sci U S A. 2021;118(32). Epub 2021/08/06. doi: 10.1073/pnas.2100369118. PubMed PMID: 34349021; PubMed Central PMCID: PMCPMC8364173.

2. Crepin S, Harel J, Dozois CM. Chromosomal complementation using Tn7 transposon vectors in Enterobacteriaceae. Appl Environ Microbiol. 2012;78(17):6001-8. Epub 2012/06/19. doi: 10.1128/AEM.00986-12. PubMed PMID: 22706059; PubMed Central PMCID: PMCPMC3416591.

3. Cho SH, Szewczyk J, Pesavento C, Zietek M, Banzhaf M, Roszczenko P, et al. Detecting envelope stress by monitoring beta-barrel assembly. Cell. 2014;159(7):1652-64. Epub 2014/12/20. doi: 10.1016/j.cell.2014.11.045

S0092-8674(14)01522-0 [pii]. PubMed PMID: 25525882.

4. Hussein NA, Cho SH, Laloux G, Siam R, Collet JF. Distinct domains of Escherichia coli IgaA connect envelope stress sensing and down-regulation of the Rcs phosphorelay across subcellular compartments. PLoS Genet. 2018;14(5):e1007398. Epub 2018/06/01. doi: 10.1371/journal.pgen.1007398. PubMed PMID: 29852010; PubMed Central PMCID: PMCPMC5978795.

5. Mirdita M, Schutze K, Moriwaki Y, Heo L, Ovchinnikov S, Steinegger M. ColabFold: making protein folding accessible to all. Nat Methods. 2022;19(6):679-82. Epub 2022/06/01. doi: 10.1038/s41592-022-01488-1. PubMed PMID: 35637307; PubMed Central PMCID: PMCPMC9184281.

6. Lee J, Patel DS, Stahle J, Park SJ, Kern NR, Kim S, et al. CHARMM-GUI Membrane Builder for Complex Biological Membrane Simulations with Glycolipids and Lipoglycans. J Chem Theory Comput. 2019;15(1):775-86. Epub 2018/12/12. doi: 10.1021/acs.jctc.8b01066. PubMed PMID: 30525595.

7. Konovalova A, Perlman DH, Cowles CE, Silhavy TJ. Transmembrane domain of surface-exposed outer membrane lipoprotein RcsF is threaded through the lumen of β-barrel proteins. Proceedings of the National Academy of Sciences of the United States of America. 2014;111(41):E4350-E8. doi: 10.1073/pnas.1417138111. PubMed PMID: WOS:000342922000013.

8. Jo S, Kim T, Iyer VG, Im W. CHARMM-GUI: a web-based graphical user interface for CHARMM. J Comput Chem. 2008;29(11):1859-65. Epub 2008/03/21. doi: 10.1002/jcc.20945. PubMed PMID: 18351591.

9. Lee J, Cheng X, Swails JM, Yeom MS, Eastman PK, Lemkul JA, et al. CHARMM-GUI Input Generator for NAMD, GROMACS, AMBER, OpenMM, and CHARMM/OpenMM Simulations Using the CHARMM36 Additive Force Field. J Chem Theory Comput. 2016;12(1):405-13. Epub 2015/12/04. doi: 10.1021/acs.jctc.5b00935. PubMed PMID: 26631602; PubMed Central PMCID: PMCPMC4712441.

10. William L. Jorgensen JC, and Jeffry D. Madura. Comparison of simple potential functions for simulating liquid water. J Chem Phys. 1983;79, 926. doi: https://doi.org/10.1063/1.445869.

11. Hopkins CW, Le Grand S, Walker RC, Roitberg AE. Long-Time-Step Molecular Dynamics through Hydrogen Mass Repartitioning. J Chem Theory Comput. 2015;11(4):1864-74. Epub 2015/11/18. doi: 10.1021/ct5010406. PubMed PMID: 26574392.

12. Eastman P, Swails J, Chodera JD, McGibbon RT, Zhao Y, Beauchamp KA, et al. OpenMM 7: Rapid development of high performance algorithms for molecular dynamics. PLoS Comput Biol. 2017;13(7):e1005659. Epub 2017/07/27. doi: 10.1371/journal.pcbi.1005659. PubMed PMID: 28746339; PubMed Central PMCID: PMCPMC5549999.

13. Ulrich Essmann LP, and Max L. Berkowitz. A smooth particle mesh Ewald method. J Chem Phys 1995;103, 8577. doi: https://doi.org/10.1063/1.470117.
